# Advances of Various Heterogeneous Structure Types in Molecular Junction Systems and Their Charge Transport Properties

**DOI:** 10.1002/advs.202202399

**Published:** 2022-08-17

**Authors:** Jaeho Shin, Jung Sun Eo, Takgyeong Jeon, Takhee Lee, Gunuk Wang

**Affiliations:** ^1^ KU‐KIST Graduate School of Converging Science and Technology Korea University Seoul 02841 Korea; ^2^ Department of Physics and Astronomy Institute of Applied Physics Seoul National University Seoul 08826 Korea; ^3^ Department of Integrative Energy Engineering Korea University Seoul 02841 Korea; ^4^ Center for Neuromorphic Engineering Korea Institute of Science and Technology Seoul 02792 Korea; ^5^ Department of Chemistry Rice University 6100 Main Street Houston Texas 77005 United States

**Keywords:** charge transport mechanism, molecular electronics, molecular heterojunction, molecule‐2D material heterostructures, molecule‐inorganic heterostructures, molecule‐organic heterostructures, three‐terminal molecular heterojunction

## Abstract

Molecular electronics that can produce functional electronic circuits using a single molecule or molecular ensemble remains an attractive research field because it not only represents an essential step toward realizing ultimate electronic device scaling but may also expand our understanding of the intrinsic quantum transports at the molecular level. Recently, in order to overcome the difficulties inherent in the conventional approach to studying molecular electronics and developing functional device applications, this field has attempted to diversify the electrical characteristics and device architectures using various types of heterogeneous structures in molecular junctions. This review summarizes recent efforts devoted to functional devices with molecular heterostructures. Diverse molecules and materials can be combined and incorporated in such two‐ and three‐terminal heterojunction structures, to achieve desirable electronic functionalities. The heterojunction structures, charge transport mechanisms, and possible strategies for implementing electronic functions using various hetero unit materials are presented sequentially. In addition, the applicability and merits of molecular heterojunction structures, as well as the anticipated challenges associated with their implementation in device applications are discussed and summarized. This review will contribute to a deeper understanding of charge transport through molecular heterojunction, and it may pave the way toward desirable electronic functionalities in molecular electronics applications.

## Graphical abstract

The authors present recent advances to develop a wide spectrum of molecular heterostructures, as well as their prospects and applicability. Various molecular heterostructures and their novel electrical characteristics, along with their charge transport mechanisms are presented. In addition, the potential applicability, merits, and perspectives, as well as the anticipated challenges associated with their implementation in electronic device applications are discussed.

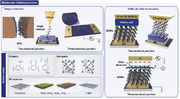

## 1 Introduction

Over the last half century, various types of functional devices have been built using semiconductor heterojunctions; these have laid the groundwork for the development of the modern electronics industry.^[^ ^1^, ^2^, ^3^, ^4^, ^5^, ^6^ ^]^ An electronic heterostructure is defined as a junction of two materials with distinct energy bands in which the charge transport properties can be effectively controlled and improved under the influence of external electrical and optical sources, to achieve desirable electronic functionality. For example, a silicon diode can be constructed using a heterojunction of n‐ and p‐type Si.^[^ ^7^ ^]^ Diverse heterojunctions, including Si/SiGe, Si/GaAs, and Si/InGaAs, have also been developed; these exhibit high carrier mobility and low‐power operation.^[^ ^8^, ^9^, ^10^ ^]^ In addition to this electronic application range, modern solar cells, sensors, and light‐emitting diodes (LEDs) have been extensively explored and proposed, based upon various forms of heterojunctions (composed of diverse emerging nanomaterials) as well as conventional semiconductor heterostructures.^[^ ^11^, ^12^, ^13^, ^14^, ^15^, ^16^, ^17^, ^18^, ^19^, ^20^ ^]^ Moreover, for enhanced organic phototransistors (OPTs), incorporating additional materials (e.g., colloidal quantum dots, surface plasmonic nanomaterials, or perovskites) into the channel represents a well‐established method for enhancing photonic functionalities.^[^ ^21^, ^22^, ^23^, ^24^, ^25^, ^26^ ^]^

**Figure 1:**
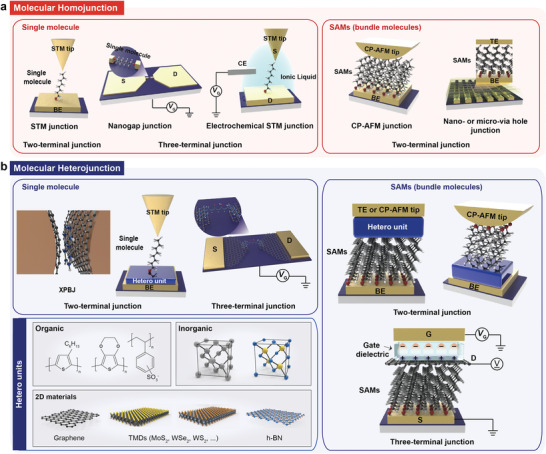
a) Diverse molecular homojunction platforms in (left) single‐molecule based and (right) molecular‐SAM (or bundle) based molecular junctions under two‐terminal and three‐terminal device structures. Two‐terminal single molecular junctions and molecular‐SAM (or bundle) junctions based on STM, and CP‐AFM and nano‐ or micro via hole junction, respectively. Three‐terminal single molecular junction platforms based on nanogap and STM junction platforms. b) Diverse molecular heterojunction platforms in (left) single‐molecule based and (right) molecular‐SAM (or bundle) based molecular junctions under two‐terminal and three‐terminal device structures consisting of (left bottom) various hetero units (e.g., organic, inorganic, and 2D materials). Two‐terminal single molecular junctions and molecular‐SAM (or bundle) junctions based on cross plane break junction (XPBJ) and STM, and CP‐AFM and nano‐ or micro via hole junction platforms, respectively. Three‐terminal single molecular junction based on graphene‐nanogap.

Similarly, the field of molecular electronics has recently attempted to diversify the electrical characteristics and device architectures of various types of molecular heterojunction, by exploiting a combination of molecules and other nano or organic materials. Historically, molecular junction types, referred to as molecular homojunctions, have been classified into two‐ and three‐terminal junctions with a single molecule or molecular self‐assembled monolayer (SAM) (i.e., bundle of molecules), as shown in **Figure** **1**a.^[^ ^27^, ^28^, ^29^ ^]^ In fact, since two‐ and three‐terminal junctions are the main passive and active device architectures in modern electronic goods, junction platforms for molecular electronics have also been designed in these shapes. The two‐terminal molecular junction structure employs only a sub‐1 nm single molecule or molecular SAMs placed between the top and bottom conductive electrodes (TE and BE, respectively); this facilitates a unique charge transport mechanism that primarily depends upon the molecular structure itself and the contact geometry (Figure 1a). For decades, numerous research groups have fabricated two‐terminal molecular homojunctions using scanning tunneling microscopy (STM),^[^ ^30^, ^31^, ^32^, ^33^, ^34^ ^]^ conducting probe atomic force microscopy (CP‐AFM)^[^ ^35^, ^36^, ^37^, ^38^, ^39^, ^40^, ^41^, ^42^ ^]^ and nano or micro via hole patterning^[^ ^43^, ^44^, ^45^, ^46^, ^47^, ^48^, ^49^, ^50^, ^51^, ^52^, ^53^ ^]^ through metal evaporation. These junction structures have been recognized as basic platforms for the characterization of single molecule or molecular SAMs and their intrinsic quantum transport properties. Meanwhile, three‐terminal molecular junction structures employ a solid back gate at the molecular nanogap junction (where a single molecule is laterally connected at the nanogap) or an electrochemical gate (through ionic liquid) to the side of the single molecular junctions using STM (the left of Figure 1a).^[^ ^54^, ^55^, ^56^, ^57^ ^]^ In this type of molecular junction, the transport barriers between the single molecular orbital level and Fermi level (*E*_F_) of the electrodes can be modulated by applying a gate‐induced electric field, thereby altering the overall charge transport across the molecule.

Because both types of molecular junction allow for the use of countless molecular structure variables and facilitate the ultimate scale in high‐density integration, this field has attracted considerable attention for more than 50 years and has been extensively investigated using a variety of functional molecular species and junction structures. This endeavor has resulted in the realization of a variety of electronic functions in these molecular junctions, including diode,^[^ ^58^, ^59^, ^60^, ^61^ ^]^ memory,^[^ ^62^, ^63^, ^64^, ^65^ ^]^ photoswitching,^[^ ^66^, ^67^, ^68^, ^69^ ^]^ thermoelectric effects,^[^ ^70^, ^71^, ^72^, ^73^ ^]^ and field‐effect transistors.^[^ ^55^, ^74^ ^]^ However, these conventional types of molecular junctions suffer from several challenges, including tailored electrical functionalities and limited functional molecular components, operational stabilities, and junction yields.^[^ ^75^, ^76^, ^77^, ^78^, ^79^ ^]^ For example, various electrical functions and high performance may be restricted because charge transport is primarily determined by the energy levels of the molecular species. Furthermore, the general design principles for a molecular structure capable of exhibiting desirable electrical characteristics have not yet been concretely established, which could limit the applicability of molecular junctions. Electrical stability has also been a longstanding issue for these types of molecular‐scale junctions, because of their extremely low junction yield and high levels of operational unreliability and thermal fluctuation.^[^ ^75^, ^76^, ^77^, ^78^, ^79^ ^]^ In particular, the charge transport current in typical two‐ and three‐terminal molecular junctions varies significantly, despite minor variations in the contact geometry and surface morphology; this limits their utility as reliable electrical device units beyond charge transport studies.^[^ ^80^, ^81^, ^82^, ^83^ ^]^ Consequently, developing a novel strategy and methods for overcoming these issues has emerged as the primary challenge in this field.

Recently, with the development of fabrication technology and well‐tailored nanomaterial synthesis, molecular junction architectures designed to tackle these issues have been suggested and are becoming more diverse. Among these recent advances, various molecular heterojunctions in the two‐ and three‐terminal forms have been reported (Figure 1b). Numerous attempts have been made to implement molecular heterostructures for novel electrical functionalities, operational stability, and tunability, thereby expanding their applicability.^[^ ^84^, ^85^, ^86^, ^87^, ^88^, ^89^ ^]^ Essentially, a molecular heterojunction is composed of molecules and hetero units such as 2D or 3D semiconductors or nanomaterials; this facilitates the tailoring of various electronic functions and operational stabilities that are extremely difficult to achieve when using only molecules. In particular, 2D or 3D semiconductors with a certain bandgap structure can align hetero energy bands with a molecular band structure, thereby changing the charge transport pathways according to the electrical and junction variables.^[^ ^90^, ^91^, ^92^, ^93^, ^94^, ^95^, ^96^ ^]^ This could lead to a diversity of electronic functionalities in the molecular heterojunction form, as well as a well‐established junction structure that can be stably operated.

Several progress reviews in the field of molecular electronics have been reported in terms of device platform, yield, and electrical functionalities.^[^ ^27^, ^28^, ^29^, ^97^, ^98^, ^99^, ^100^, ^101^, ^102^, ^103^, ^104^ ^]^ For example, Xiang et al. provided an overview of molecular junctions at the level of the device platform and charge transport phenomena.^[^ ^27^ ^]^ Various molecular junction platforms and theoretical simulations were described in detail. Thereafter, they introduced the concept of integrating molecular functionalities into electronic circuits such as diodes, transistors, memory, and sensors. Jeong et al. presented various device platforms for high‐yield molecular junctions and their functionalities; here, distinctive electronic functionalities originated from the molecular structure and contact properties.^[^ ^29^ ^]^ They discussed an interlayer strategy and distinctive device fabrication procedure for high‐yield molecular devices. Lu et al. reviewed various micro/nanofabrication strategies for creating molecular junctions with a single molecule that enables desirable functionality.^[^ ^101^ ^]^ They discussed the difficulties and opportunities associated with the fabrication of single molecular devices. Lastly, Xie et al. presented various single molecular junction platforms for monitoring and manipulating the physical and chemical behavior of molecular components.^[^ ^104^ ^]^ They presented a summary of various dynamic investigations conducted at single‐molecular scale and discussed the open issues in this field. All of these reviews, however, have mainly addressed the traditional molecular junction structures (in this review, we refer to them as molecular homojunction systems) based on the metal‐molecule‐metal junction type first proposed by Aviram and Ratner in 1974.^[^ ^105^ ^]^ In contrast to previous reviews, we discuss recent advances made in efforts to develop a wide spectrum of heterogeneous structures in molecular junctions, as well as their prospects and applicability. In particular, various types of two‐ and three‐terminal molecular heterojunction structures and their novel electrical characteristics, along with their charge transport mechanisms are presented sequentially. In addition, the potential applicability and merits of molecular heterojunction structures, as well as the anticipated challenges and issues associated with their implementation in electronic device applications are discussed and summarized. This review departs from previous reviews in this field in terms of addressing heterogeneous structures in molecular junctions and their potential, and also offers a fresh perspective on how to advance this field further.

The remainder of this paper is organized as follows. After the introduction (Section 1), the molecular heterojunctions in two‐terminal devices are discussed, along with their functionalities (Section 2). Various types of molecular heterojunctions are introduced (according to the constituents of the molecular heterojunction systems): molecule‐organic heterostructures (Section 2.1), molecule‐inorganic heterostructures (Section 2.2), and molecule‐2D semiconductor heterostructures (Section 2.3). In addition, as novel types of molecular transistors, three‐terminal lateral and vertical molecular‐graphene heterojunction configurations employing a single and bundle of molecules are introduced and discussed. (Section 3). Finally, the merits of the molecular heterojunction structures, as well as the anticipated challenges and issues associated with their implementation (which are critical to the success of next‐generation electronic devices for practical applications) are discussed (Section 4); this is followed by a brief conclusion (Section 5).

## 2 Two‐Terminal Molecular Heterojunctions

In Section 2, we discuss various two‐terminal molecular heterojunction structures and the potential transport mechanisms that govern those systems. Two‐terminal molecular heterojunctions are composed of heterostructured molecules and 2D or 3D semiconductors or nanomaterials.^[^ ^84^, ^85^, ^86^, ^87^, ^88^, ^89^, ^90^, ^91^, ^92^, ^93^, ^94^, ^95^, ^96^ ^]^ The heterostructure is sandwiched vertically between the top and bottom electrodes. Diverse two‐terminal molecular heterojunctions have been investigated using STM,^[^ ^92^, ^93^ ^]^ atomic force microscopy (AFM),^[^ ^94^, ^95^, ^96^ ^]^ and microscale via hole techniques.^[^ ^84^, ^85^, ^86^, ^87^, ^88^, ^89^, ^90^, ^91^ ^]^ In these junctions, by incorporating 2D transition metal dichalcogenides (TMDs) or 3D (organic, Si, or Ge) semiconductors, various hetero band alignments can be made; these can activate different charge transport pathways, depending on the molecular or semiconductor energy band structures, the molecular dipole moments and their directions, and the voltage polarities. In addition, by incorporating a functional nanomaterial (e.g., a conducting organic polymer or doped (undoped) graphene), the device stability, yield, and junction transparency can be significantly increased; the last of these allows the device to be used as a molecular photoswitching junction platform.

### 2.1 Molecule‐Organic Heterostructures

When molecular SAMs are formed on a metal electrode (Au, Pt, Ag, or Cu), their permanent dipole moment can modify the metal work function (*Φ*_M_) by varying the interfacial potential drop.^[^ ^90^, ^91^, ^106^, ^107^, ^108^, ^109^, ^110^, ^111^, ^112^, ^113^, ^114^ ^]^ *Φ*_M_ can be increased or decreased according to the directions of the molecular dipole moment, and its magnitude is determined by the difference in electronegativity between the metal–SAM interface and molecular backbone structure.^[^ ^106^, ^107^, ^108^, ^109^, ^110^, ^111^ ^]^ Furthermore, the pinning effect at the Fermi level is alleviated because the SAM‐modified metal electrode has an almost dangling‐bond‐free interface.^[^ ^106^, ^107^ ^]^ And, the non‐covalent or physical contact can be formed between molecules and organic semiconductor. Because of this effect, the interfacial transport barrier in the molecule‐organic semiconductor heterostructure can be determined by the offset between the Fermi level of the SAM‐modified metal and the conduction (or valence) band of the subsequent organic layer. In this sense, this junction structure creates an asymmetric hetero‐band alignment, facilitating the enhancement of rectifying features or optoelectronic properties via the selective blocking of electrons or holes. This method has frequently been employed to enhance the electrical properties of organic semiconductor devices.^[^ ^107^, ^111^, ^113^, ^114^, ^115^ ^]^ However, the molecular SAMs have only been used for interfacial barrier modulation, to improve the electrical performance of the organic electronic device; furthermore, the junction size is much larger than the molecular scale. Nevertheless, this strategy could offer a promising method and technological ground for implementing and understanding a functional molecular‐scale heterojunction, as will be discussed later.

**Figure 2:**
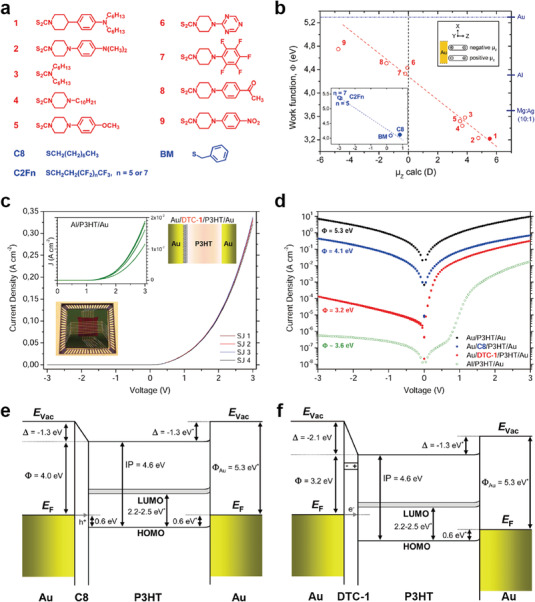
a) Molecular structures of the investigated DTC (red) and thiolate (blue) derivatives. b) Dependence of the work function *Φ* (from UV photoelectron spectroscopy (UPS) measurements) on the dipole moment *µ*_z_ (from density functional theory calculations) for the nine DTC derivatives investigated in this study. The inset shows the graph for thiolate monolayers. As a reference, relevant values of *Φ* for commonly used metals and alloys are shown on the right ordinate. c) Diode characteristics in junctions implementing the Au/DTC‐1/P3HT/Au structure (the layer structure is shown in the upper right inset). In the left inset, *J*–*V* characteristics from the Al/P3HT/Au structure are presented for comparison. The standard deviation of *J* (at +3 V) from the mean is 2% for Au/DTC‐1/P3HT/Au junctions and 2% for Al/P3HT/Au junctions. The lower inset shows the crossbar structure mounted on a chip carrier. d) Semilog plot showing the averaged *J*–*V* curves for Au/SAM/P3HT/Au and Al/P3HT/Au junctions, as well as for Au/P3HT/Au reference junctions (black data points). Energy band diagram illustrating the band alignment in e) Au/C8/P3HT/Au and f) Au/DTC‐1/P3HT/Au junctions, as obtained from UPS measurements. Reproduced with permission.^[^ ^90^ ^]^ Copyright 2014, American Chemical Society.

Ford et al. reported two‐terminal molecule‐organic semiconductor heterojunctions and their electrical characteristics with respect to different molecular SAM species.^[^ ^90^ ^]^ They used a p‐type organic semiconductor, poly(3‐hexylthiophene) (P3HT). Several dithiocarbamate (DTC)‐ and thiol‐based molecular species were used, as shown in **Figure** **2**a. They demonstrated that these molecular SAMs layers featuring highly robust dipoles and chemically bonded interfaces were capable of shifting the work function (*Φ*) of Au and Ag to ≈3.2 eV, as shown in Figure 2b. They investigated the modified *Φ* values by molecular SAMs based on low kinetic energy photoemission threshold (secondary photoemission cutoff) in the ultraviolet photoelectron spectroscopy (UPS). Depending on the *z*‐direction magnitude of the molecular dipole moment (*µ*_z_), *Φ* can be tuned over the range of ≈1.6 eV. A linear dependence of *Φ* on *µ*_z_ was also observed. To investigate the effect of the molecular SAMs and their dipoles on the device performance, electrical measurements of Au/molecular SAMs/P3HT/Au heterojunctions were performed (Figure 2c,d). Note that the molecular SAMs can be easily formed by chemical bonding between Au and sulfur atoms. Since the P3HT is known to form crystals with the *π*‐stacking direction in the plane of the coated film and has a specific and small dipole moment, it is highly probable that the interactions between the SAMs and P3HT are the dipole–dipole interactions with van der Waals force. As shown in Figure 2d, the Au/DTC‐1/P3HT/Au heterojunction (red) had a much higher rectification ratio (RR) (of ≈2 × 10^3^) compared to the Au/P3HT/Au junction (black) (RR ≈ 1) (at ±3 V). However, in the Au/C8/P3HT/Au heterojunction (blue), the rectifying feature was not observed, though the current density (*J*) was approximately one order of magnitude lower than that of the Au/P3HT/Au junction (green). In the Al/P3HT/Au junction (green), the RR was found to be ≈2 × 10^4^ and *J* was lower by three orders of magnitude compared to the Au/P3HT/Au junction. These findings can be explained on the basis of the following two considerations: 1) the effect of the molecular tunneling barrier and 2) the effect of the asymmetric hetero‐energy level alignments at both contacts on the transport conduction. First, the attenuation of *J* was primarily associated with the presence of molecular SAMs sandwiched between the Au and P3HT. The DTC‐1 and C8 molecules formed a tunneling barrier at the Au/P3HT interface, resulting in a decrease in *J*, as shown in Figure 2d.^[^ ^116^ ^]^ In the case of the Al/P3HT/Au junction, the native oxide on the Al surface (AlO*_x_*) (with a thickness of ≈3 nm) caused a reduction in *J*.^[^ ^117^ ^]^ Second, the effect arising from the asymmetric hetero‐energy level alignment in the junction can be considered. Figures 2e,f show the hetero‐energy band diagrams for the Au/C8/P3HT/Au and Au/DTC‐1/P3HT/Au heterojunctions, respectively. The Au/C8/P3HT/Au junction had similar interfacial hole barriers because of the pinning effect (fixed at ≈0.6 eV height) between the Au and P3HT.^[^ ^118^ ^]^ This results in symmetrical electrical characteristics. However, for the Au/DTC‐1/P3HT/Au junction, the DTC‐1 monolayer can tune the work function of Au to 3.2 eV, closer to the lowest unoccupied molecular orbital (LUMO) level of P3HT. As a result, this junction establishes different contact barriers between the two interfaces, which rectifies the electrical properties (Figure 2d,f).

**Figure 3:**
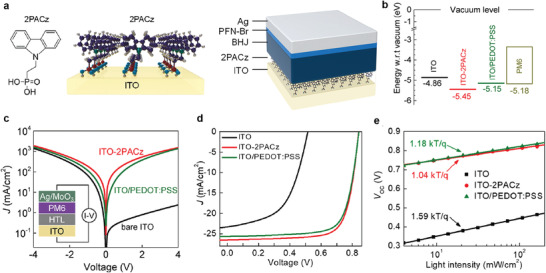
a) Chemical structure of 2PACz and schematic illustration of the ITO‐2PACz electrode (left), as well as a schematic of the standard cell architectures employed. b) Work function of ITO, ITO‐2PACz, ITO/PEDOT:PSS, and HOMO level of PM6 measured via photoelectron spectroscopy in air (PESA) technique. c) Dark current through a hole‐only device based on ITO, 2PACz, and PEDOT:PSS. The inset shows the schematic of the hole‐only cell's architecture. d) *J*–*V* curves of PM6:N3 solar cells based on ITO, ITO‐2PACz, and ITO/PEDOT:PSS. e) Light intensity dependence of *V*_OC_ for the same cells. Reproduced with permission.^[^ ^91^ ^]^ Copyright 2020, American Chemical Society.

Another example of molecule‐organic heterostructures has also been found in organic photovoltaic device applications. Lin et al. reported organic photovoltaic (OPV) devices using 2PACz (where 2PACz is [2‐(9H‐Carbazol‐9‐yl)ethyl]phosphonic acid) molecules as a hole‐selective transport layer (HTL) functionalized directly onto an indium tin oxide (ITO) anode (**Figure** **3**a).^[^ ^91^ ^]^ Subsequently, PM6:N3 was spin‐coated on 2PACz molecular SAM, which could form a van der Waals contact between them. They demonstrated that the work function of the 2PACz‐functionalized ITO electrode (−5.45 eV) was significantly deeper than that of the bare ITO (−4.86 eV) and ITO/PEDOT:PSS (−5.15 eV) electrodes, owing to the molecular dipole moment (Figure 3b). They used photoelectron spectroscopy in air (PESA) to verify the work function of ITO electrode before and after 2PACz functionalization. In their result, the hole‐injection barrier at the interface of the PM6 organic layer was negligible, resulting in symmetric *J*–voltage (*V*) characteristics in the forward and reverse bias regions (Figure 3c). The representative *J*–*V* characteristics for all three OPV devices using ITO, ITO‐2PACz, and ITO/PEDOT:PSS are shown in Figure 3d. Remarkably, when 2PACz SAM was used as HTL, the power conversion efficiency (PCE) of 16.6%, the short‐circuit current (*J*_SC_) of 26.53 mA cm^−2^, fill factor (FF) of 74.5%, and series resistance (*R*_s_) of 2.47 Ω cm^2^ were improved compared to the bare ITO junction and ITO‐PEDOT:PSS junction. This is because the ITO‐2PACz electrode had a lower interface resistance than the ITO or ITO/PEDOT:PSS electrodes, resulting in a greater FF and overall PCE. Figure 3e shows the dependence of the open‐circuit potential (*V*_OC_) on the light intensity for the ITO, ITO‐2PACz, and ITO/PEDOT:PSS electrodes. The slope of ITO‐2PACz electrode showed 1.04 *kT*/*q*, significantly lower than that for the ITO electrode and the ITO/PEDOT:PSS electrode. A larger slope indicates that trap‐assisted recombination was significantly more involved.^[^ ^119^ ^]^ Therefore, ITO‐2PACz‐based OPV devices can reduce trap‐assisted recombination, leading to higher PCE and longer carrier lifetimes. Thus, the functionalization of 2APCz onto ITO can improve the functionality and stability of OPV devices by engineering the interface between the ITO and PM6 organic layers.

**Figure 4:**
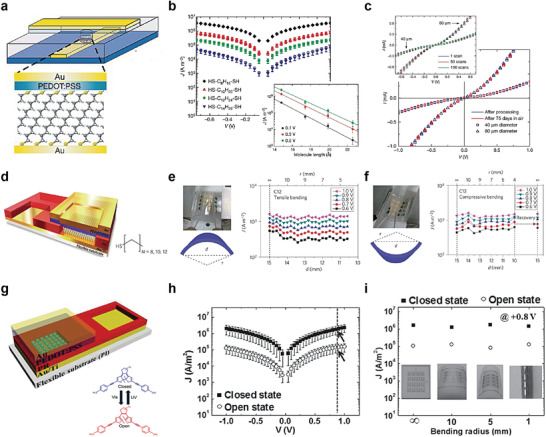
a) Completed molecular junction composed of Au/PEDOT:PSS/SAM/Au molecular heterojunctions. b) The error bars give the standard deviation upon averaging over at least 17 devices. Inset, *J* (on a log scale) at 0.1, 0.3, and 0.5 V biases as a function of the molecular length. This indicates that the transport is achieved by through‐bond tunneling. c) *I*–*V* characteristics of molecular junctions based on dodecane dithiol with diameters of 40 µm (squares) and 80 µm (triangles), as measured both directly after processing (blue) and after storing under ambient conditions for 75 days (red). The inset shows three *I–V* measurements out of 100 consecutive sweeps of a molecular junction stored under ambient conditions for 75 days with diameters of 40 µm (squares) and 80 µm (triangles). Reproduced with permission.^[^ ^84^ ^]^ Copyright 2006, Nature Publishing Group. d) Schematics of a flexible molecular device. The layers (from bottom to top) are as follows: flexible substrate, bottom gold/titanium electrode, molecular layer or photoresist (PR) (for electrical isolation), PEDOT:PSS layer, and top gold electrode. Optical images and schematic diagrams of e) the tensile and f) compressive substrates. *J* values of the C12 devices at five different voltages as a function of *d* (bottom axis) and *r* (top axis) under e) tensile and f) compressive bending. Reproduced with permission.^[^ ^85^ ^]^ Copyright 2012, Nature Publishing Group. g) Completed diarylethene molecular devices on a flexible substrate (PI substrate) and the chemical structures of the diarylethene molecules, which can be converted from an open (low conductance) state to a closed (high conductance) state via exposure to UV and visible light, respectively. h) *J–V* curves for the closed and open state diarylethene molecular devices on a flexible substrate under flat conditions. i) *J* values measured at 0.8 V for closed and open states under flat substrate condition (bending radius = ∞) and different bending conditions (bending radii = 10, 5, and 1 mm). Inset photos show the molecular devices under different bending radius configurations. Reproduced with permission.[^86^ ^]^ Copyright 2014, Wiley‐VCH.

Organic polymers such as PEDOT:PSS have been utilized as units of molecular‐organic heterostructures for high yields of molecular junctions.^[^ ^67^, ^84^, ^85^, ^86^, ^120^, ^121^, ^122^, ^123^, ^124^ ^]^ In this junction structure, molecular SAMs and metal form covalent bonds through a chemical anchoring group, whereas molecular SAMs and PEDOT:PSS form non‐covalent bonds through van der Waals force. Akkerman et al. first suggested a molecule‐PEDOT:PSS heterostructure in molecular junctions.^[^ ^84^ ^]^ Not only did interfacial PEDOT:PSS serve as a conductive electrode, it also prevented top metal penetration through the partially defective molecular SAMs layer, which can form a metallic filament. Based on this strategy, they demonstrated a reliable solid‐state molecular device with >95% yield (**Figure** **4**a). Note that a typical molecular junction formed with Au/molecule/Au has a <2% device yield. Various alkanedithiol molecular species were used for the molecular SAMs, including octanedithiol (denoted as HS—C_8_H_16_—SH), octanedithiol (HS—C_10_H_20_—SH), dodecanedithiol (HS—C_12_H_24_—SH), and tetradecanedithiol (HS—C_14_H_28_—SH). Figure 4b shows very stable *J*–*V* non‐resonant tunneling curves for all molecular junctions with the interfacial PEDOT:PSS layer; this is also consistent with previous experimental and theoretical expectations. It was also found that these tunneling transports were maintained regardless of the number of scans or junction diameters for 75 days, as shown in Figure 4c. Furthermore, this molecular heterostructure containing PEDOT: PSS can be fabricated on a flexible substrate, which can be extended to implement a solid‐state flexible molecular junction. Park et al. first developed Au/alkanethiol/PEDOT:PSS/Au junctions on a polyimide (PI) flexible substrate for use in flexible molecular‐scale electronic devices (Figure 4d).^[^ ^85^ ^]^ They demonstrated that the *J* values remained approximately constant during both tensile and compressive bending (Figure 4e,f). This indicates that the molecule‐PEDOT:PSS heterojunction can exhibit excellent operational stability and durability under various deformation conditions. Using the same junction platform, Kim et al. reported a flexible molecular photoswitching junction using diarylethene molecular SAMs (Figure 4g).^[^ ^86^ ^]^ Diarylethene molecules can have two conductance states (high or low conductance); these are controlled by exposure to the UV or visible light (bottom of Figure 4g) that can pass the PEDOT:PSS. The *J*–*V* characteristics of diarylethene molecular devices in the open and closed states are shown in Figure 4h, where the closed state has a higher *J* than the open state. This is because when the open state of diarylethene is exposed to UV light, the central ring of the molecular structure is closed, leading to activation of the transport pathway along the *π*‐conjugated molecular structure. In contrast, when the closed state of diarylethene is exposed to visible light, the *π*‐conjugation of the current path is broken, leading to a decrease in *J* (Figure 4g). In this system, light passes through the thin Au/PEDOT:PSS layers and approaches the molecules. Similar to the results shown in Figure 4e,f, the *J* (*V* = 0.8 V) values of both the open and closed states could be retained without any deterioration, depending on the bending radii (Figure 4i).

From the perspective of electronic and optoelectronic organic device applications, molecular SAMs can be adopted to tune the interfacial transport barrier in the device architecture. The molecule‐organic semiconductor heterojunction can establish asymmetric (or symmetric) band alignments and improve electrical characteristics. In particular, the molecule‐PEDOT:PSS heterojunctions can significantly improve the yield of molecular electronic devices with excellent operational stability, reproducibility, and reliability. Moreover, this heterojunction can be useful for designing flexible molecular photoswitching electronic devices. Consequently, the molecular junctions combined with organic semiconductors or organic conductor can not only aid in fabrication of reliable solid‐state molecular heterojunctions, but also to enhance our understanding of the charge transport through molecular layer due to the reproducible electrical characteristics observed.

### 2.2 Molecule‐Inorganic Heterostructures

When molecular SAMs are directly formed on an inorganic 3D semiconductor (Si or GaAs), the depletion region at the heterojunction interface can be largely varied in response to the polarity of the applied voltage.^[^ ^86^, ^92^, ^93^, ^125^, ^126^, ^127^, ^128^, ^129^ ^]^ In this heterostructure, larger charge carriers flow in one direction, enhancing the rectifying characteristics. Furthermore, when a semiconductor with a direct band gap is irradiated with light of an appropriate wavelength, electrons and holes are generated separately in the conduction and valence bands, respectively.^[^ ^93^ ^]^ This photogenerated electron–hole pair can be separated across the direction of band bending in the semiconductor (owing to the molecular dipole moment), even when the voltage is not applied. This strategy can improve the photo‐response electrical characteristics.

**Figure 5:**
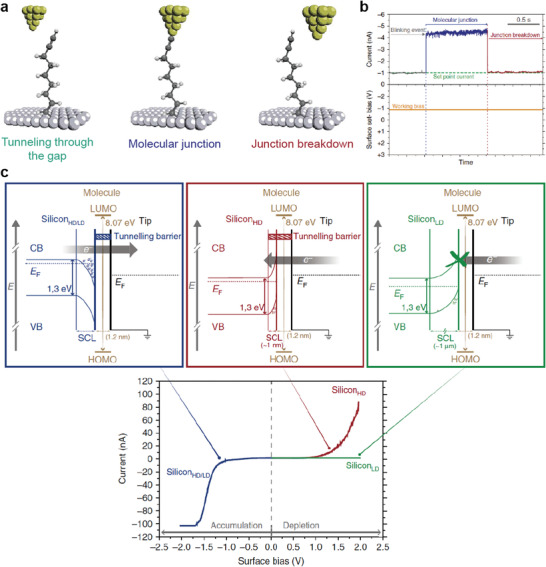
a) Schematic of the STM junction experiment describing the formation and breakdown of the Au‐nonadiyne‐Si junction. b) Example of the “blink” captured when a 1,8‐nonadiyne molecule bridges the two electrodes at −0.8 V. Current jumps (blink) above the set‐point tunneling current appear when the nonadiyne molecule bridges the gap between the gold and silicon electrodes. c) Energy band diagram representations of the Au‐nonadiyne‐Si junction under charge carrier accumulation conditions (applied negative sample voltages) in the Si electrode (left panel) and under depletion conditions (applied positive sample voltage) for the Si_HD_ (central panel) and Si_LD_ (right panel). Changing the starting bias does not affect the shape of the IV curves. Reproduced with permission.^[^ ^92^ ^]^ Copyright 2017, Nature Publishing Group.

For example, Aragonès et al. reported electrical charge transport in a two‐terminal single molecule‐Si heterojunction using a scanning tunneling microscopy break‐junction (STM‐BJ) approach.^[^ ^92^ ^]^ They formed 1,8‐nonadiyne SAMs with highly ordered structures on n‐type Si surfaces via a hydrosilylation reaction.^[^ ^130^, ^131^, ^132^ ^]^ The 1,8‐nonadiyne SAMs on the n‐type Si surface could inhibit surface oxidation and make stable chemical bonding with the Au STM tip, resulting in the formation of an n‐type Si‐1,8‐nonadiyne/Au junction (i.e., forming single molecular heterojunction) (**Figure** **5**a). To verify the highly ordered molecular structure on n‐type Si surface, they conducted the X‐ray photoelectron spectroscopy (XPS) measurement and confirmed that there is no silicon oxide or sub‐oxides, and presence of C 1s signals corresponding to —C≡C— groups and silyated olefin (Si—C=C). This indicates that the molecular SAMs on Si substrate was chemically well‐formed. They used the blinking approach to produce mechanically stable heterojunctions, in which an initial set‐point tunneling current was delivered under a fixed bias voltage, whereas the Au STM tip approached the 1,8‐nonadiyne terminated n‐type Si surfaces (Figure 5a,b). Based on this junction, *I*–*V* characteristics were investigated using highly n‐doped Si (denoted as n‐Si_HD_) and low n‐doped Si (denoted as n‐Si_LD_) in the absence of light. In the n‐Si_HD_‐1,8‐nonadiyne/Au junction, when a negative bias was applied to the n‐Si_HD_ surface, electrons accumulated in the space charge region (SCR) (Figure 5c, left panel). In this case, electrons can flow through the transport barrier, which is primarily determined by the molecular tunneling barrier. Conversely, when a positive bias was applied, the SCR decreased to ≈1 nm (depletion region). In this case, the transport barrier is determined by both the molecular tunneling barrier and SCR width. (Figure 5c, central panel). Nevertheless, the *I*–*V* characteristics were slightly asymmetric because the effect of the 1 nm‐SCR and its band bending was limited in terms of affecting on the overall charge transport. In contrast, in the n‐Si_LD_‐1,8‐nonadiyne/Au junction, the SCR increased to ≈1 µm when a positive bias was applied. This thicker SCR can suppress the transport current from the Au tip; as a result, they achieved a large asymmetry and rectifying characteristics, with an RR of ≈4000 (at ±1.5 V) (Figure 5c, right panel). Note that the junction without 1,8‐nonadiyne SAMs showed random and unpredictable rectifying characteristics, regardless of the doping level of the Si substrate. Therefore, the two‐terminal molecule Si (n‐Si_LD_) heterojunction structure could attain very high and reproducible rectifying *I*–*V* characteristics.

**Figure 6:**
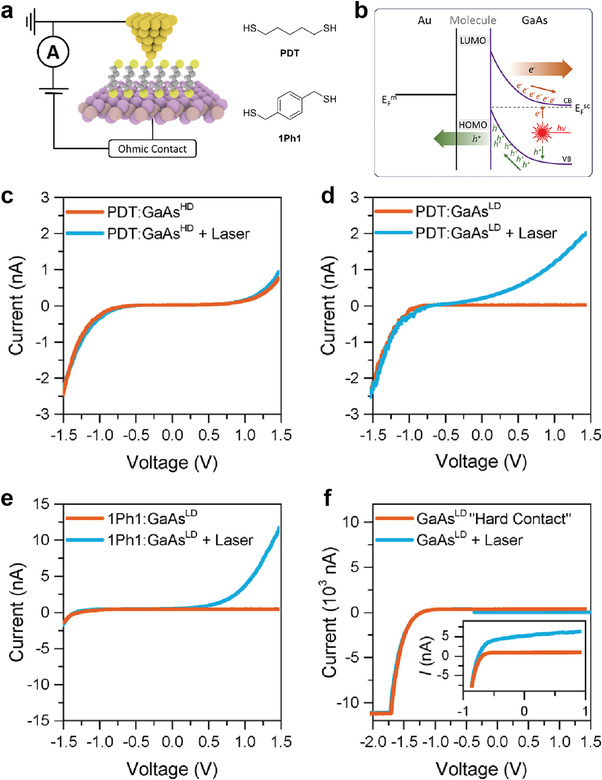
a) Device schematic and structures of the molecular wires used in this study. b) Schematic band diagram for the illuminated metal‐molecule‐semiconductor junction under reverse bias. Junction *I–V* characteristics for c) PDT on GaAs^HD^, d) PDT on GaAs^LD^, e) 1Ph1 on GaAs^LD^, and f) “hard contact” between Au and GaAs^LD^ wafers fabricated by crashing the tip several µm into a freshly etched GaAs surface. The inset shows an enlargement of the low‐current area between −1 and 1 V. Reproduced with permission.^[^ ^93^ ^]^ Copyright 2017, American Chemical Society.

In addition, molecule‐inorganic semiconductor heterostructures can be employed to improve the electrical characteristics of the photoresponse. Vezzoli et al. designed a photodiode heterojunction consisting of molecular SAMs and GaAs, using the STM‐BJ approach.^[^ ^93^ ^]^ They formed SAMs of 1,5‐pentanedithiol (PDT) and 1,4‐phenylene(dimethanethiol) (1Ph1) with highly ordered structures on n‐type GaAs surfaces, and chemical bonding can be formed by Au‐STM tip‐S bonding (**Figure** **6**a). Note that they also used different n‐doped GaAs substrates, including highly n‐doped GaAs (denoted as GaAs^HD^) and low n‐doped GaAs (denoted as GaAs^LD^). As illustrated in Figure 6b, when the laser (632.8 nm wavelength) illuminated the heterojunction, the photogenerated electrons and holes moved in opposite directions, owing to the band bending generated by the molecular SAMs. More specifically, photogenerated holes were transferred to the Au electrode via the molecular tunnel barrier (the hole‐injection barrier), whereas electrons were transferred to the n‐doped GaAs. In the GaAs^HD^‐PDT‐Au tip junction, the *I*–*V* characteristics were nearly symmetric and constant, regardless of the laser illumination (Figure 6c). This symmetric electrical transport can also be understood by the same principle as that observed in the n‐Si_HD_‐1,8‐nonadiyne/Au junction (Figure 5c). In addition, because the SCR is very thin, laser illumination had a negligible effect on the *I*–*V* characteristics. In contrast, in the GaAs^LD^‐PDT‐Au tip junction, they exhibited a rectifying *I*–*V* characteristic with RR > ≈1000 under dark conditions and achieved a high photocurrent under laser illumination in the positive bias region (Figure 6d). Because the SCR of GaAs^LD^ was 2.7 times thicker than that of GaAs^HD^, a significantly larger photocurrent could be generated.

In the case of 1Ph1 SAMs, the photocurrent can be further enhanced because 1Ph1 has a smaller band gap and hole injection barrier than PDT. Figure 6e shows the electrical characteristics of the GaAs^LD^‐1Ph1‐Au junction in the presence and absence of laser illumination. Under dark conditions, the RR was found to be ≈10^2^, lower than that obtained when PDT SAMs were used. However, under laser illumination, the photocurrent response was significantly increased compared to the case of the PDT SAMs. This is because more photogenerated holes could be transported through the reduced hole‐injection barrier.^[^ ^133^, ^134^ ^]^ Figure 6f shows the photoelectric response for the “Hard Contact” GaAs^LD^ junction where the Au STM tip was directly contacted to a GaAs^LD^ semiconductor without a molecular layer. Compared with the heterojunction implementing molecular SAMs, this junction exhibited a very low photoelectric response. Thus, by incorporating molecular SAMs into this junction and adjusting the band bending via molecular dipoles, the optoelectronic properties could be enhanced.

To summarize, in this molecule‐inorganic heterostructure system, molecular SAMs placed directly onto inorganic 3D semiconductors can determine the degree of band bending in the semiconductor at the interface, SCR, and charge‐injection barriers; this can significantly affect the rectifying characteristics and photocurrent response. In particular, since the molecular heterojunction formed by the STM‐BJ method is a single molecular heterojunction, both reports indicate that the rectifying and photoswitching properties can be enhanced even at the single‐molecular scale. In this sense, the molecule‐inorganic semiconductor heterojunction structure may offer a promising strategy to improve the electronic and optoelectronic properties of molecular junctions at the single molecular scale.

### 2.3 Molecule‐2D Material Heterostructures

#### 2.3.1 Molecule‐Graphene Heterostructure

The molecular‐2D materials heterojunctions were initially developed in the form of metal/molecular SAMs/multi‐layer graphene (MLG) structures, to increase the yield of molecular devices whilst maintaining a molecular‐scale junction.^[^ ^87^, ^88^, ^135^, ^136^, ^137^, ^138^ ^]^ In this two‐terminal vertical heterojunction, graphene (a one‐atomic‐thick 2D material of covalently bonded carbon atoms with outstanding mechanical and electrical properties) was utilized as both a top conductive electrode and a preventing layer that blocked top metal penetration through molecular SAMs. Moreover, since the graphene is sp^2^ hybridized honeycomb lattice structure, the *π*–*π* stacking interaction between single molecule and graphene can give the single molecular cross‐plane junction (i.e., two‐terminal lateral heterojunction) to explore the charge transport through molecular cross‐plane. This one‐atomic‐thick graphene could form a heterojunction on a molecular scale, much smaller than other types of molecular heterostructures (i.e., molecule‐organic and molecule‐inorganic heterostructures).

**Figure 7:**
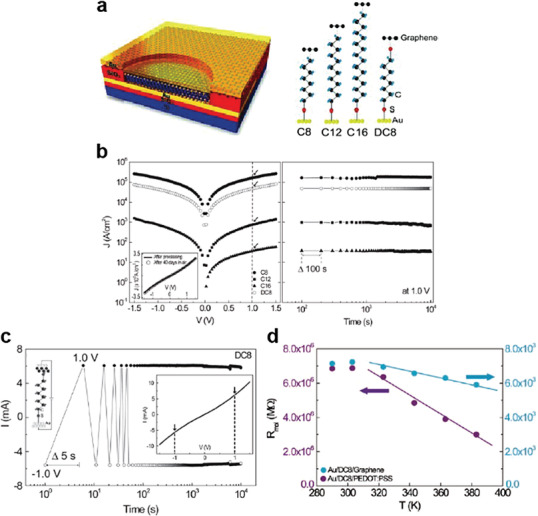
a) Schematic of completed molecular junction device and four types of studied molecular systems along with their chemical structures: C8, C12, C16, and DC8. b) (Left) The *J–V* characteristics for representative C8, C12, C16, and DC8 molecular devices. The inset shows a DC8 device as measured after fabrication (solid line) and after storage under ambient conditions for 40 *d* (open circles). (Right) Retention characteristics of the molecular junctions in terms of the measured *J* at 1.0 V for 10^4^ s (measurement interval: Δ*t* = 100 s). c) Retention characteristics of the cross‐measured positive current (measured at +1.0 V) and negative current (measured at –1.0 V) for 10^4^ s with an interval Δ*t* = 5 s. d) The *R*_mol_ values for Au/DC8/PEDOT:PSS and Au/DC8/graphene devices as a function of increasing temperature. Reproduced with permission.^[^ ^87^ ^]^ Copyright 2011, Wiley‐VCH.

Wang et al. developed an Au/molecular SAM/MLG/Au heterojunction for reliable solid‐state molecular‐scale devices (**Figure** **7**a).^[^ ^87^ ^]^ Various alkanethiol molecular species were used for the molecular SAMs, including octanethiol (C8), dodecanethiol (C12), hexadecanethiol (C16), and octanedithiol (DC8) (the right of Figure 7a). The MLG interlayer prevented the formation of Au filaments brought about by the top Au electrode penetration; this enhanced the yield of molecular devices by ≈90%. To create the two‐terminal vertical molecule‐graphene heterojunction, molecular SAMs were directly formed on an Au substrate confined to a specific area, and graphene was then transferred onto the molecular SAMs to establish van der Waals interactions between the molecular SAMs and graphene electrode. Figure 7b shows *J*–*V* curves for C8, C12, C16, and DC8 molecules with the MLG electrode. Depending on the molecular length of the alkanemonothiol (C8, C12, and C16), *J* exponentially decreases, indicating that off‐resonant tunneling was the main conduction mechanism.^[^ ^52^ ^]^ The C8 junction, on the other hand, exhibited a greater *J* than the DC8 junction, in contrast to the conventional two‐terminal metal‐molecule‐metal junction. Because MLG did not form a chemisorbed contact with —SH or —CH_3_, the higher conductance of the C8 junction compared to that of the DC8 junction originated from the shorter contact length in the C8 junction. The inset of Figure 7b shows the *J*–*V* curves for the C8 molecule, as measured immediately after device fabrication and after storage under ambient conditions for 40 days. These *J*–*V* curves were almost similar, indicating that the molecular junction was preserved by the MLG electrode without any deterioration. The right of Figure 7b shows the operational stability obtained by *J* (*V* = 1.0 V) as a function of time, retaining the value of *J* for 10^4^ s with measurement interval Δ*t* = 100 s. Figure 7c shows that the DC8 molecular devices also had good retention, realized by the cross‐measured retention of *J* at *V* = 1.0 V and −1.0 V for 10^4^ s with a measurement interval Δ*t* = 5 s. These results indicate the excellent durability, operational stability, and device lifetime of the molecular junction with MLG electrodes. Figure 7d shows the resistance per single molecule (*R*_mol_) for the DC8 molecular junction with different top electrodes (PEDOT:PSS or graphene), depending on the temperature (from 290 to 383 K).^[^ ^139^ ^]^ The *R*_mol_ value for the DC8 molecular junction with PEDOT:PSS rapidly decreased under increasing temperature (323–383 K). This is because molecular desorption or removal of the remaining moisture from the hydrophilic PEDOT:PSS/molecule interface can produce a phase change in the SAM, resulting in a rapid decrease in *R*_mol_.^[^ ^139^ ^]^ On the other hand, *R*_mol_ for the DC8 molecular junction with graphene slowly decreased under increasing temperature. This result indicates that graphene‐based molecular devices have a greater thermal stability than molecule/PEDOT:PSS molecular heterojunctions. Therefore, this study suggests that molecule/MLG heterojunctions are a reliable platform for the application of molecular junctions.

**Figure 8:**
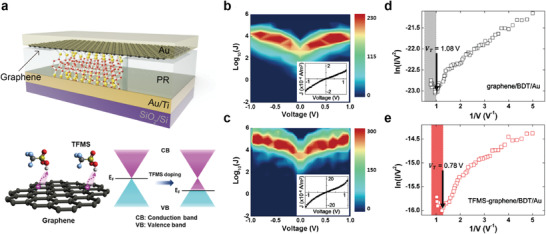
a) (Top) Schematic of the molecular junction in this study (not scaled) and (bottom) schematic of the TFMS‐doped graphene (left) and its corresponding shift in the energy band diagram (right). 2D *J–V* plots of the working molecular junctions for the b) graphene/BDT/Au and c) TFMS‐graphene/BDT/Au junctions. Representative TVS data of the d) graphene/BDT/Au and e) TFMS‐graphene/BDT/Au junctions. Reproduced with permission.^[^ ^88^ ^]^ Copyright 2017, American Chemical Society.

Following that report, and because of the distinct feature of graphene in electrochemical doping, a molecule/doped graphene heterojunction system was also studied. Jang et al. reported a molecular heterojunction structure based on a chemically p‐doped graphene film prepared using trifluoromethanesulfonic acid (CF_3_SO_3_H, denoted as TFMS).^[^ ^88^ ^]^ The benzene‐1,4‐dithiol (denoted as BDT) SAMs were sandwiched between the multi‐layer p‐doped or pristine graphene top electrode and Au bottom electrode (**Figure** **8**a). TFMS‐treated p‐doped graphene has a higher work function (≈5.23 eV) than pristine graphene (≈4.4 eV), which reduces the interfacial molecular barrier height.^[^ ^140^ ^]^ They investigated the raster canning of Raman spectra to verify the increase in the hole concentration after TFMS treatment. The Raman spectra of the TFMS‐treated graphene film showed upshifts of the G and 2D peaks with negligible D bands, indicating TFMS‐induced p‐type doping effect on graphene without generating significant defects. As a result, the average *J* increased by approximately one order of magnitude for the molecular junction with TFMS‐treated p‐doped graphene, compared to that prepared with pristine graphene (Figure 8b,c). This tendency was consistent with the difference in the transition voltage spectroscopy (TVS) between the two molecular heterojunctions, which estimates the effective barrier height, as shown in Figure 8d,e.^[^ ^123^, ^141^, ^142^, ^143^ ^]^ Figures 8d,e show the Fowler–Nordheim (F–N) plot (ln (*I*/*V* ^2^) vs 1/*V*) for the molecular junction with pristine graphene or TFMS‐treated p‐doped graphene. The TVS (or transition voltage, *V*_T_) was obtained from the inflection points of the F–N plots. The averaged *V*_T_ for the molecular junction with pristine graphene or TFMS‐treated p‐doped graphene were ≈1.08 V and ≈0.78 V, respectively. This result indicates that the enhanced charge transport mainly originates from the barrier‐height‐lowering effect induced by the increase in the work function of TFMS‐treated p‐doped graphene. Therefore, the charge transport phenomena of molecule‐graphene heterojunctions can be tuned by the interfacial doping engineering of graphene.

**Figure 9:**
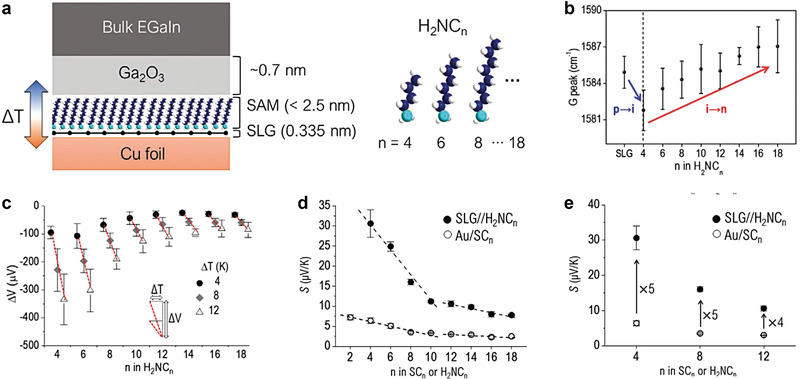
a) (Left) Schematic describing the structure of large‐area thermoelectric junction and (right) molecular structures. b) The *G* peak (cm^−1^) shift in Raman spectra for H_2_NC*_n_* SAMs on SLG. The “p,” “i,” and “n” indicate p‐type doped, intrinsic, and n‐type doped SLGs, respectively. c) Trends of Δ*V* as a function of various Δ*T* for the SAMs. d) Comparison of length dependence of Seebeck coefficient (*S*, µV K^−1^) for noncovalent (SLG/H_2_NC*_n_*) and covalent (Au/SC*_n_*) junctions. e) Enhanced *S* values in the noncovalent interface as compared to the covalent one. Reproduced with permission.^[^ ^73^ ^]^ Copyright 2021, Wiley‐VCH.

This molecule‐graphene heterostructure can also enhance the thermoelectric effect in the molecular junction. Recently, Park et al. reported the thermoelectric molecular heterojunction comprising eutectic gallium‐indium (EGaIn)/n‐alkylamines/single‐layer graphene (SLG) (**Figure** **9**a).^[^ ^73^ ^]^ They enhanced the Seebeck coefficient (*S*) in noncovalent contact between SLG for a bottom electrode and n‐alkylamines SAMs (H_2_NC*_n_* where *n* = 4, 6, 8, …, 18; right of Figure 9a). The SLG on copper foil (SLG/Cu) was incubated in a 18 mm solution of H_2_NC*_n_* with methanol and tetrahydrofuran (THF) mixed solvent to form H_2_NC*_n_* SAMs on SLG. The noncovalent interaction between amine anchor of SAMs and SLG allows reversible adsorption and desorption for self‐repair, leading to closed packed monolayers without chemical contact on SLG.^[^ ^144^, ^145^, ^146^, ^147^ ^]^ Owing to the inevitable water treatment and handling of sample in air conditions, the large‐area graphene synthesized by the chemical vapor deposition (CVD) method tends to be p‐type doped compared to exfoliated graphene (Figure 9b).^[^ ^148^ ^]^ However, when the n‐alkylamines SAMs were treated on the SLG, the G peak position in the Raman spectra was blueshifted with respect to the position of pristine SLG (Figure 9b). This was attributed to n‐type doping of SLG by the amine anchor.^[^ ^149^, ^150^ ^]^ In addition, when the alkyl chain length increased, the degree of blueshift of the G peak gradually increased, which confirmed the densely packed molecular SAMs structure.

Figure 9c demonstrates that the Δ*V* of all SAMs increased as Δ*T* increased, resulting in positive *S* values. Moreover, the value of deviation in Δ*V* increased with increasing Δ*T* and shortening the length of H_2_NC*_n_* molecular SAMs. This is because the lateral intermolecular interaction in longer molecular SAMs was stronger than that in shorter molecular SAMs, leading to densely packed molecular structures in longer molecular SAMs.^[^ ^151^ ^]^ To examine the effect of noncovalent contact on thermopower, they compared *S* values for Gr/H_2_NC*_n_*/Ga_2_O_3_/EGaIn and Au/SC*_n_*/Ga_2_O_3_/EGaIn junctions (Figure 9d). Interestingly, all S values for noncovalent contact junctions (e.g., Gr/H_2_NC*_n_*/Ga_2_O_3_/EGaIn) were higher (approximately a factor of five) than that for covalent contact junctions (e.g., Au/SC*_n_*/Ga_2_O_3_/EGaIn junctions) (Figure 9e). This is because the noncovalent contact between SLG and amine anchor can efficiently suppress the phonon transport, leading to smaller thermal conductance by an order of magnitude than that in the covalent contact between Au and thiol anchor.^[^ ^152^, ^153^ ^]^ In addition, both molecular junctions exhibited the same crossover point, *n* = 10 (Figure 9d). This was attributed to the densely packed molecular structures in longer molecular SAMs. In the molecular SAMs of *n* < 10, the molecular SAM structures were relatively low packing density compared to that of *n* ≥ 10. The surface coverage on SLG and the packing stabilization energy in H_2_NC*_n_* SAMs vary noticeably at *n* = 10,^[^ ^144^, ^145^, ^154^ ^]^ which accounts for the transition in the slope of plots shown in Figure 9d. Therefore, the thermopower in saturated molecules can be enhanced by noncovalent contact properties between molecular SAMs and SLG electrode.

With these demonstrations in the two‐terminal vertical molecule‐graphene heterojunction (Figures 7, 8, 9), it is possible to conclude that this strategy can increase device yield, demonstrate stable electrical characteristics and a greater thermoelectric effect, and provide an optoelectronic switchable molecular heterojunction platform while preserving the molecular‐scale active region.

**Figure 10:**
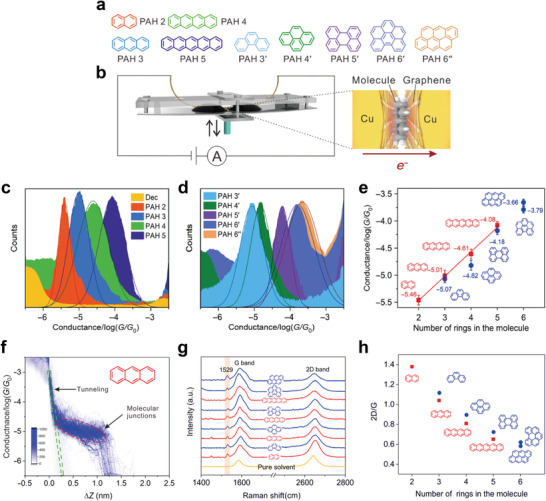
a) Chemical structures of the polycyclic aromatic hydrocarbons (PAHs) that sandwiched between two graphene electrodes. b) Schematic of the cross‐plane break junction (XPBJ) technique and the device structure of the studied graphene‐molecule‐graphene heterojunctions. 1D conductance histograms generated from ≈1000 traces for graphene‐molecule‐graphene heterojunctions of c) the linear PAHs and d) the nonlinear PAHs. e) The single‐molecule conductance of each molecular heterojunctions, plotted as a function of the number of benzene rings. The error bar is determined from the chip‐to‐chip variation of three independent experiments. f) The 2D conductance‐distance histogram for the molecular heterojunctions with PAH 3. The graphene rectangular dashed frame represents tunneling in decane, and the red elliptical dashed frame represents molecular junctions. g) Raman spectrum of the graphene electrode pair that experienced XPBJ operation in the presence of pure solvent (the yellow curve), and the Raman spectra of the molecular heterojunctions fabricated by the XPBJ method. h) The ratio of the intensities of the G and 2D peaks as a function of the number of benzene rings. Reproduced with permission.^[^ ^155^ ^]^ Copyright 2020, American Association for the Advancement of Science.

Contrary to many previous studies through two‐terminal vertical molecule‐graphene heterojunctions, charge transport across the plane of a single molecule has been demonstrated experimentally only rarely. Recently, Zhao et al. fabricated a graphene‐single molecule‐graphene heterojunction based on cross‐plane break junction (XPBJ) technique.^[^ ^155^ ^]^ They investigated the electrical characteristics of the graphene‐single molecule‐graphene heterojunction consisting of a family of polycyclic aromatic hydrocarbons (PAHs) with different number of rings (**Figure** **10**a,b). By repeatedly controlling the gap between two graphene sides in a molecular solution, the conductance histograms of linear and nonlinear PAH molecular junctions with varying numbers of PAH ring structures were analyzed (Figure 10c,d). Due to the increase in cross‐plane area, the conductance through cross‐plane junctions increased as the number of PAH rings increased (Figure 10e). This behavior differs from the typical molecular junction, in which the conductance decreases exponentially as the molecular barrier width (or length) increases.^[^ ^156^, ^157^ ^]^ Therefore, the fact that the conductance increased as the length of the molecules increased indicated that the molecules are located between two graphene electrodes while lying flat and that the charge is transferred via the cross‐plane molecular route. Figure 10f depicts the construction of a 2D conductance–distance histogram for graphene‐PAH molecule–graphene heterojunctions, allowing the determination of a range of charge transport length across the nanogap. It could be considered that the conductance plateau in approximately between 0.3 and 1.3 nm arises from molecular junctions.^[^ ^158^, ^159^ ^]^ The most probable plateau length lied in 0.87 to 1.08 nm regardless of molecular length, where the distance was much smaller than the molecular lengths of the PAH molecules. This indicated that the charge transfers through cross‐plane molecular junctions. Raman spectroscopy^[^ ^160^ ^]^ was utilized to demonstrate such a molecular configuration between graphene electrodes (Figure 10g). There was a significant pack at 1529 cm^−1^ attributed to the adsorption of PAH molecules on the graphene surface. In contrast, this signal disappeared on pristine graphene surface. In addition, the intensities ratio of the G and 2D peaks that is determined by the electron concentration of graphene was decreased with longer molecules, indicating that the degree of charge transport between cross‐plane molecules and graphene electrode increases with longer molecules (Figure 10h). These results are consistent with the path analysis for the graphene‐molecule‐graphene heterojunctions, as shown Figure 10e. Note that similar experiments and analyses were also conducted on the cross‐plane molecule‐graphene heterojunction platform employing the fullerene molecule.^[^ ^161^ ^]^ This two‐terminal lateral single molecule‐graphene heterojunction can be used as a new type of single molecular junction platform to understand the intrinsic quantum transport at the single molecular scale by decreasing the uncertainty in controlling the gap between metal electrodes.

**Figure 11:**
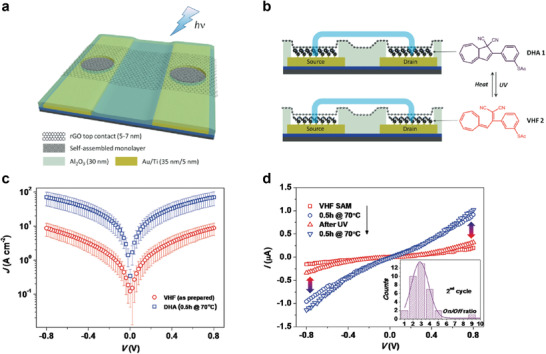
a) Schematic view of molecular test bed with rGO thin film as transparent top contact. b) Schematic cross section of the molecular junction and molecular structure of DHA 1 and VHF 2, to illustrate the thermos‐optical switching. c) *J–V* characteristics of VHF 2 (as prepared) and DHA 1 (70 °C for 0.5 h) isomers self‐assembled in the molecular junctions. The data were averaged from at least 24 junctions of the same batch. d) Representative bidirectional conduction switching characteristics induced by alternating heating and UV irradiation. UV irradiation typically does not fully recover the low‐conducting state of VHF 2 and therefore results in a decrease of the on/off ratio (inset). Reproduced with permission.^[^ ^89^ ^]^ Copyright 2013, Wiley‐VCH.

In addition, molecule‐(reduced) graphene oxide (GO or rGO) heterojunctions were also suggested for transparent and photo switchable molecular junction. Owing to the superior processing properties of GO or rGO over graphene, they can be used more straightforwardly and conveniently for large‐scale device fabrication.^[^ ^89^, ^162^, ^163^, ^164^, ^165^ ^]^ Li et al. recently reported a large‐scale light/heat‐switchable Au/molecular SAM/rGO heterojunction (**Figure** **11**a,b).^[^ ^89^ ^]^ The junction was 2 or 4 µm in diameter. The special double‐junction layout allowed the SAMs to be switched whilst simultaneously facilitating electrical measurements across the molecular junction. That study used DHA 1/VHF 2 isomers as molecular SAMs formed on the Au bottom electrode (right side of Figure 11b). This isomer facilitated reversible conformational changes between DHA 1 and VHF 2 under light illumination and heat treatment.^[^ ^166^, ^167^, ^168^, ^169^ ^]^ Because the top rGO film was transparent and had high in‐plane conductivity (compared with the molecular component), it enabled conductive top‐electrode contact and interconnection between SAMs in series (without additional top‐metal contact) under light illumination. To verify the reversible conformational changes under light illumination and heat treatment, the XPS was employed to determine the properties of the SAMs. The obvious changes were not observed after treatments and the S 2p signals showed only one doublet with S 2p_3/2_ attributed to an S atom chemically bonded to Au without oxidation signal. Figure 11c shows the *J*–*V* characteristics for DHA 1 and VHF 2 molecular junctions with an average conductance ON/OFF ratio of 5–7. The VHF 2 isomer molecular junction was shown to have a larger *J* than that of DHA 1. This is because the increased backbone flexibility of the VHF 2 isomer produces a larger conformational freedom than DHA 1, varying the coupling strengths to the physisorbed contact in rGO.^[^ ^170^ ^]^ Furthermore, consecutive UV illumination and heat treatment can reversibly switch the conductance of this molecular heterojunction, allowing the ON/OFF ratio to exceed 3 (Figure 11d). Thus, the molecule‐rGO heterojunction could be used for light/heat‐switchable solid‐state molecular junctions.

To summarize, graphene material‐based molecular heterojunctions can enhance the device yield with stable electrical characteristics and provide an optoelectronic switchable molecular heterojunction platform. Owing to van der Waals interaction between molecules and graphene, the enhanced thermoelectric effect and single molecular cross‐plane junctions can be realized where it can be utilized to deeply understand the quantum transport at the single molecular scale. In addition, the energy band alignment at the graphene‐molecule interfaces could be modulated by the chemical doping of graphene, tailoring the charge transport characteristics. In the case of rGO‐molecule heterojunctions, this can be useful for large‐scale molecular heterojunction device platforms. Lastly, owing to the unique electronic structure of graphene, the molecule‐graphene heterojunction structure can be extended to a three‐terminal transistor, permitting gate‐tunable electrical characteristics. This is discussed in Section 3.

#### 2.3.2 Molecule‐2D Semiconductor Heterostructures

TMDs, which are 2D sheets with a thickness of three atoms (e.g., MoS_2_, WSe_2_, and WS_2_) have attracted significant attention owing to their good mobility, excellent ON/OFF ratio of 10^7^–10^8^, high exciton binding energy, and strong optical absorption.^[^ ^171^, ^172^, ^173^, ^174^, ^175^, ^176^, ^177^, ^178^ ^]^ Unlike graphene, molecule‐TMD heterojunctions can form various hetero‐band structures with an alignment of two bandgap structures between the top and bottom conductive electrodes.^[^ ^179^, ^180^ ^]^ In particular, depending on the different electrical and junction variables (e.g., the orientation of the molecular dipole moment, molecular species, molecular length, and types of 2D TMDs), their band alignment can be tuned differently.^[^ ^94^, ^95^, ^96^ ^]^ Therefore, this heterojunction structure offers a promising means to engineer and unfold novel electrical functionalities by modulating the dominant charge transport pathways and interfaces; this cannot be accomplished using typical molecular SAMs or 2D TMD junctions.

**Figure 12:**
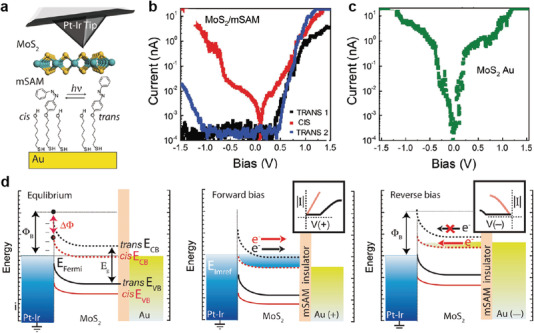
a) Schematics of the heterostructure measurement. Monolayer and multi‐layers of MoS_2_ are mechanically exfoliated on top of a mixed‐SAM (mSAM) made of spacer molecules and photoswitchable (black arrow) azobenzene derivatives (1:1) on gold. b) Representative *I–V* characteristics in semilogarithmic scale for MoS_2_/mSAM heterostructure before (*trans*_1_) and after UV (*cis*) and white light exposure (*trans*_2_). c) *I–V* characteristic for 1_L_ MoS_2_/Au. d) Schematics of the metal‐semiconductor rectification barrier between the grounded Pt–Ir tip and the MoS_2_/mSAM Au heterostructure. The transport channel of MoS_2_/*cis*‐mSAM is closer to the electrodes’ Fermi levels at equilibrium, and transport readily occurs at any bias. The misaligned transport channel for the MoS_2_/trans‐mSAM heterostructure at equilibrium requires a larger bias for transport at the corresponding polarities. Reproduced with permission.^[^ ^94^ ^]^ Copyright 2015, Wiley‐VCH.

Recently, Margapoti et al. reported a photoswitching diode using conductive AFM (C‐AFM) on a mixed‐self‐assembled monolayer (mSAM)/mono (1_L_)‐MoS_2_ heterostructure.^[^ ^94^ ^]^ They used the (4‐(1‐mercapto‐6‐hexyloxy)‐azobenzene) (HS‐C_6_AZO) as a photo‐switching molecule and 6‐(2‐mercapto)‐1‐hexanol as a spacer molecule. The 1_L_‐MoS_2_ was directly exfoliated on top of the molecular SAMs on the Au substrate; then, a Pt–Ir coated AFM tip was used to measure the electrical characteristics (**Figure** **12**a). Due to the dangling‐bond‐free interface between molecular SAMs and 1L MoS_2_, van der Waals interaction can exist and affect the energy band of MoS_2_ according to molecular conformation. The photoisomerization of HS‐C_6_AZO can be changed from *cis* to *trans* under illumination with 366 nm UV‐light for 30 min, and it can be changed from *trans* to *cis* under white light illumination for 2 h.^[^ ^181^, ^182^ ^]^ As shown in Figure 12b, in the case of the *trans‐*mSAM junction/1_L_‐MoS_2_ junction, rectifying characteristics can be observed, with an RR of >10^3^. In contrast, for the *cis*‐mSAM/1_L_‐MoS_2_ junction, *I–V* characteristics tended to be symmetrical. Symmetric *I*–*V* characteristics were observed in the junction without mSAM, meaning only the 1_L_‐MoS_2_ junction exhibited symmetric *I*–*V* characteristics (Figure 12c). This is because the *trans*‐mSAM modified Au has a decreased work function (Δ*Φ*_B_ = 1.03 ± 0.10 V) compared to *cis*‐mSAM modified Au (Δ*Φ*_B_ = 0.67 ± 0.10 V), resulting in a greater interface barrier at 1_L_‐MoS_2_/Au (Figure 12d). The modified work function by molecular SAMs can be obtained from difference in the contact potential of the heterostructures using scanning Kelvin probe microscopy (SKPM). In the case of the *cis*‐mSAM/1_L_‐MoS_2_ junction, electrons can be easily transported at any bias polarity, because the conduction band of 1_L_‐MoS_2_ is closely aligned to the Fermi level of the two electrodes. However, in the case of the *trans*‐mSAM/1_L_‐MoS_2_ junction, the conduction band of 1_L_‐MoS_2_ lies far from the bottom Au electrode, and electrons in the reverse bias region can be blocked by the Schottky barrier at the 1_L_‐MoS_2_/Au interface, leading to a higher RR than the *cis*‐mSAM/1_L_‐MoS_2_ junction (the right of Figure 12d). This heterojunction system can further enhance the photoswitching characteristics of the *trans*‐ and *cis*‐mSAM.

**Figure 13:**
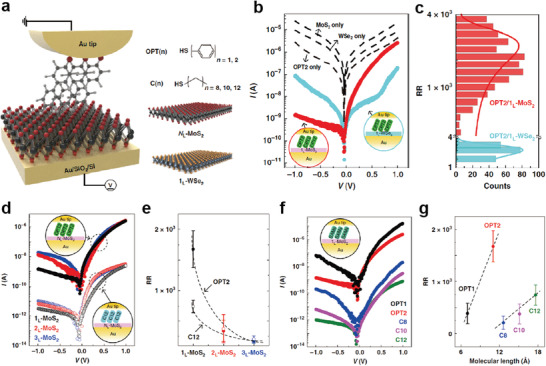
a) Schematic of molecular heterojunction composed of OPT2 and 1_L_‐MoS_2_ stacked on an Au/SiO_2_/Si substrate, as obtained using the C‐AFM technique (left). Five different molecular species (OPT (*n* = 1, 2) and C (*n* = 8, 10, 12)) and two different 2D semiconductor types (*N*_L_ (1_L_, 2_L_, or 3_L_)‐ MoS_2_, and 1_L_‐WSe_2_) are shown (right). b) Representative *I–V* characteristics of the junctions without OPT2 or TMDs (dotted black line), OPT2/1_L_‐WSe_2_ (solid cyan circle), and OPT2/1_L_‐MoS_2_ (solid red circle) junctions. c) Statistical histograms of RR for OPT2/1_L_‐MoS_2_ and OPT2/1_L_‐WSe_2_ junctions. The line curves denote fitting results from the Gaussian function. Note that the total numbers of OPT2/1_L_‐WSe_2_ and OPT2/1_L_‐MoS_2_ junctions are 200 and 1100, respectively. d) Representative *I–V* characteristics for the Au/OPT2 (or C12)/*N*_L_‐MoS_2_ (*N*_L_ = 1_L_, 2_L_, or 3_L_)/Au junction. e) RR plots of OPT2/*N*_L_‐MoS_2_ and C12/*N*_L_‐MoS_2_ junctions as a function of the number of MoS_2_ layers. f) Representative *I–V* characteristics for OPT(*n*) (*n* = 1 or 2)/1_L_‐MoS_2_ and C(*n*) (*n* = 8, 10, or 12)/1L‐MoS2 junctions. g) RR plots of OPT(*n*)/1_L_‐MoS_2_ and C(*n*)/1‐MoS_2_ junctions as a function of molecular length. Reproduced with permission.^[^ ^95^ ^]^ Copyright 2020, Nature Publishing Group.

Similarly, Shin et al. reported interesting electrical characteristics for molecule‐2D TMD heterostructure junctions using non‐functional and standard molecular SAMs.^[^ ^95^ ^]^ **Figure** **13**a shows a molecular heterojunction system composed of Au/molecular SAM/TMDs/Au; this was investigated using C‐AFM. Five non‐functionalized molecules that differed in terms of molecular length and backbone structure (i.e., benzene‐1‐monothiol (denoted as OPT1), biphenyl‐4‐monothiol (OPT2), 1‐octanemonothiol (C8), 1‐decanemonothiol (C10), and 1‐dodecanemonothiol (C12)) were used as molecular monolayers. As a 2D semiconductor, *n*‐type *N*_L_‐MoS_2_ with different layers (mono (1_L_‐), bi (2_L_‐), and tri (3_L_‐)) and *p*‐type 1_L_‐WSe_2_ were used as rectifying designers in molecular heterojunctions. For junctions composed of only TMDs or molecules (dashed black lines in Figure 13b), all *I*–*V* characteristics showed symmetric behavior (RR ≈ 1), owing to the single transport barrier between the Au electrodes. However, when the TMD was inserted between the bottom Au and molecular SAM interfaces, the *I*–*V* characteristics were changed to asymmetric. For the OPT2/1_L_‐MoS_2_ molecular heterojunction, RR = (1.38 ± 0.73) × 10^3^, much larger than that for the OPT2/1_L_‐WSe_2_ (RR = 2.46 ± 1.42) molecular heterojunction (Figure 13b,c). Furthermore, this heterojunction facilitated the modulation of molecular rectifying features, by controlling the number of MoS_2_ layers, molecular species, and molecular length. RR decreased under an increase in the number of MoS_2_ layers, owing to the reduction of the MoS_2_/Au Schottky barrier (Figure 13d,e).^[^ ^183^, ^184^ ^]^ Figure 13f shows the representative *I*–*V* characteristics of the molecular heterojunction system corresponding to the molecular length and molecular species. Inserting a thicker insulating layer between the metal and semiconductor can further alleviate the Fermi‐level pinning effects.^[^ ^184^, ^185^ ^]^ In this regard, inserting longer molecules at the Au‐tip/1_L_‐MoS_2_ interface further unpinned the Fermi level, resulting in an upward shift of the 1_L_‐MoS_2_ barrier. In particular, the RR of OPT2 was much larger than that of C8, despite its similar molecular length. This is due to the higher current in the positive bias, which is attributed to the smaller highest occupied molecular orbital (HOMO)–lowest unoccupied molecular orbital (LUMO) gap of the former. Consequently, RR increased for molecules with longer molecular lengths and smaller HOMO–LUMO gaps (Figure 13g). Thus, molecule‐2D semiconductor junctions with non‐functionalized molecules can improve the rectifying characteristics by adjusting the interfacial band alignment in molecular heterojunctions, and their rectifying characteristics can be tuned by engineering the band alignment between non‐functional molecules and 2D semiconductors in molecular‐scale heterojunctions.

**Figure 14:**
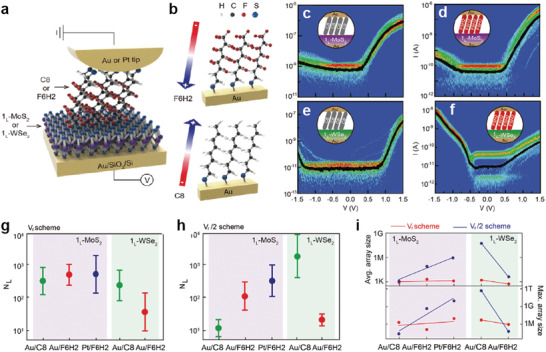
a) Schematic of the molecular heterojunction consisting of Au (or Pt)‐tip/molecule/1_L_‐2D TMD (1_L_‐MoS_2_ and 1_L_‐WSe_2_) on an Au/SiO_2_/Si substrate, which was investigated using the C‐AFM technique with *F*_L_ = 1 nN. b) Illustration of the SAM‐modified Au metal substrate with F6H2 (top) and C8 (bottom). The arrow indicates the orientation of the molecular dipole moment. Statistical heat maps of *I−V* characteristics for the molecular heterojunctions of c) Au‐tip/C8/1_L_‐MoS_2_/Au, d) Au‐tip/F6H2/1_L_‐MoS_2_/Au, e) Au‐tip/C8/1_L_‐WSe_2_/Au, and f) Au‐tip/F6H2/1_L_‐WSe_2_/Au. The estimated *N*_L_ as a function of the molecular species (C8 and F6H2), 2D TMD (1_L_‐MoS_2_ and 1_L_‐WSe_2_), and top metal electrode (Au and Pt tip) under the g) *V*_r_ and h) *V*_r_/2 schemes. i) Maximum size of the crossbar array as a function of the molecular heterojunction type under the *V*_r_ scheme (linear fit in red line) and *V*_r_/2 scheme (linear fit in blue line), based on the average *N*_L_ (top) and maximum *N*_L_ (bottom). Reproduced with permission.^[^ ^96^ ^]^ Copyright 2021, Wiley‐VCH.

In addition, Eo et al. studied the charge transport with respect to the interfacial band offsets in various molecular heterojunctions consisting of molecular SAMs with opposite dipole moments (1‐octanethiol (C8) and tridecafluoro‐1‐octanethiol (F6H2)) and different types of TMD (1_L_‐MoS_2_ and 1_L_‐WSe_2_) using the same C‐AFM technique (**Figure** **14**a).^[^ ^96^ ^]^ The surface potential drop (Δ*ϕ*) values for F6H2/Au and C8/Au junctions (as obtained by the SKPM) were 0.78 ± 0.02 eV and −0.60 ± 0.02 eV, respectively, confirming the molecular dipole moment direction of F6H2 and C8 (Figure 14b). In the molecular heterojunction with 1_L_‐MoS_2_, the RRs (defined as |*I* (*V* = 1.5 V)/*I* (*V* = −1.5 V)|) for the C8/1_L_‐MoS_2_ (RR = (4.2 ± 0.9) × 10^2^) and F6H2/1_L_‐MoS_2_ junctions (RR = (5.9 ± 0.7) × 10^2^) were positive rectification directions, regardless of the molecular dipole moment direction (Figure 14c,d). However, in molecular heterojunction with 1_L_‐WSe_2_, the RRs for C8/1_L_‐WSe_2_ and F6H2/1_L_‐WSe_2_ junctions were (3.3 ± 0.8) × 10^2^ and (2.2 ± 0.9) × 10^−2^, respectively, indicating opposite rectification directions (Figure 14e,f). This is because the energy band of 1_L_‐WSe_2_ is shifted downward or upward depending on the molecular dipole moment of C8 or F6H2; this activates different hole‐transport pathways under different voltage polarities. Based on the tunable RR designed by the hetero‐components, this molecular heterojunction could be employed as a molecular‐scale selector in an array structure. In other words, the rectifying property could mitigate the crosstalk problem induced by the activation of the leakage path in the array structure, which determines memory capacity.^[^ ^186^, ^187^, ^188^, ^189^, ^190^ ^]^ In addition, the cell footprint in the array could be reduced to the molecular scale (junction radius: ≈4 nm). The magnitudes of *N*_L_ for the *V*_r_ and *V*_r_/2 schemes were defined as resistance (*R*) (*V* = −*V*_r_)/*R* (*V* = *V*_r_) and *R* (*V* = *V*_r_/2)/*R* (*V* = *V*_r_), respectively.^[^ ^189^, ^190^ ^]^ In that study, *V*_r_ was set to 1.5 or −1.5 V (only for the F6H2/1_L_‐WSe_2_ junction). A larger *N*_L_ indicates a larger reduction in the sneak current through the unselected cells, thereby increasing the memory capacity. In the molecular heterojunction with 1_L_‐MoS_2_, the *N*_L_ in the *V*_r_/2 scheme was largely increased from (1.3 ± 0.3) × 10^1^ to (3.7 ± 0.7) × 10^2^ when the molecular species was changed from C8 to F6H2 and the top electrode was changed from Au‐tip to Pt‐tip, whereas the *N*_L_ in the *V*_r_ scheme increased slightly. However, in the molecular heterojunction with 1_L_‐WSe_2_, the *N*_L_ in the *V*_r_/2 scheme was further decreased from (2.1 ± 0.4) × 10^3^ to (2.3 ± 0.3) × 10^1^ when the molecular species was changed from C8 to F6H2 compared with the *V_r_* scheme. These phenomena indicate that *N*_L_ strongly depended on the upward (or downward) degree of band bending induced by the molecular dipole moment and metal work function. Based on the calculated *N*_L_ values for the five molecular heterojunctions, the maximum size of the crossbar array was examined according to a conventional one‐bit‐line pull‐up (one BLPU) simulation for the *V*_r_ and *V*_r_/2 schemes.^[^ ^190^, ^191^, ^192^ ^]^ Figure 14i shows the maximum size of the crossbar array in the *V*_r_ and *V*_r_/2 scheme in the molecular heterojunction with 1_L_‐MoS_2_ or 1_L_‐WSe_2_. The maximum size of the crossbar array was consistent with the trend of *N*_L_ with respect to the molecular species, metal work function, and type of 2D TMDs. Therefore, the Au/C8/1_L_‐WSe_2_/Au junction represented the optimal heterojunction combination for the maximum size of the array, achieving ≈482 Gb (Figure 14i). This study demonstrates the possibility of tailoring molecular heterojunctions for molecular‐scale selectors. Contrary to many previous studies through the molecule–graphene heterojunctions, the molecule‐2D semiconductor heterojunctions can realize hetero band alignment in molecular‐scale junctions, which can activate different charge transport pathways depending on the voltage polarity. In particular, the energy band alignment of molecule‐2D interfaces can be tuned by molecular conformational changes induced by external stimuli, molecular species and lengths, molecular dipole moments and their directions, and different types of 2D TMDs. Therefore, various types of heterojunction structures could offer promising strategies for improving electrical properties and generating novel electrical functionalities at molecular‐scale junctions.

To summarize the Section 2, the two‐terminal molecular heterojunctions based on diverse hetero units (e.g., 3D [organic and inorganic] materials and 2D materials) have the great potential to control charge transport characteristics and generate novel electrical functionalities with operational stability. In molecule‐organic material heterojunctions, they can significantly improve the device yield and produce excellent operational stabilities, owing to the existence of a subsequent hetero unit after molecular SAMs. In addition, the molecular permanent dipole moment can modify the energy band of organic materials, that can activate different charge transport pathway and generate novel electrical functionalities. However, the complexity of interface between molecule and organic materials and their junction size that is much larger than the molecular scale still remains challenges. In molecule‐inorganic materials heterojunctions, the depletion region at the heterojunction interface can be significantly modified by forming the molecular SAMs on semiconductors. Therefore, they can improve the rectifying and photo‐responsive characteristics by formation of built‐in potential. However, since the molecules are directly formed on an inorganic semiconductor, the device yield still remains challenging due to the metal penetration through molecular SAM layers. In molecule‐2D material heterojunctions, they are one of the best candidates for improving the generating electrical functionalities with high device yield. The multi‐layer graphene can sufficiently prevent the metal penetration and produce excellent operation stabilities. Moreover, the 2D TMD materials, which have certain bandgap, can activate different charge transport pathways depending on external stimuli and their energy band can be tuned by molecular permanent dipole moment. Therefore, they can offer promising ways to engineer the electrical characteristics and improve device yield and operational stabilities within ultimate scaling limit. Nevertheless, the difficulties of device fabrication in molecule‐2D materials and wafer‐scale fabrication process have still remained challenges. The details of merits and challenges of molecular heterojunctions will be discussed in Section 4.

## 3 Three‐Terminal Molecular Heterojunctions

**Figure 15:**
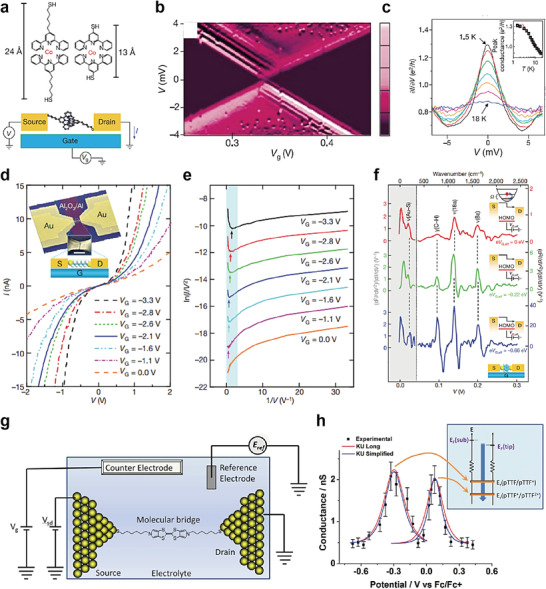
a) Molecular structures of the Co complexes and schematic diagram of the single‐molecule transistors. b) Coulomb blockade observed in devices formed from longer molecules. c) Kondo peaks as a function of voltage. The inset depicts the conductance as a function of the temperature at *V* = 0. Reproduced with permission.^[^ ^74^ ^]^ Copyright 2002, Nature Publishing Group. d) Representative *I−V* curves measured at 4.2 K for different values of *V*_G_. Inset, the device structure and schematic: S, source; D, drain; G, gate. Scale bar: 100 nm. e) Fowler–Nordheim plots corresponding to the *I−V* curves in (d), showing the transition from direct to Fowler–Nordheim tunneling with a clear gate dependence. The plots are offset vertically for clarity. The arrows indicate the boundaries between transport regimes (corresponding to *V*_trans_). f) IET spectra measured at 4.2 K for different values of *eV*_G,eff_, with vibration modes assigned. The left‐hand *y*‐axis corresponds to the grey shaded region of the spectra, and the various right‐hand *y*‐axes (with different scales) correspond to the related (color‐coded) spectra in the non‐shaded region. The vertical dotted line corresponds to *V* = 45 mV (363 cm^−1^). Significant modification in the spectral intensity and line shape for the benzene ring modes, *γ*(C—H), *ν*(18a), and *ν*(8a), was observed for different values of *eV*_G,eff_, as indicated. Insets, energy diagrams illustrating inelastic tunneling as the position of the HOMO resonance shifts through gating. Reproduced with permission.^[^ ^55^ ^]^ Copyright 2009, Nature Publishing Group. g) Schematic illustration of the electrochemical gating in a single molecule in an electrolyte. h) Conductance−sample potential relationship of pTTF. The red lines depict the long KU model, and the blue lines depict the simplified KU model. For both redox transitions, the KU model versions fit the experimental data well. The inset depicts a schematic energy level representation of the KU model for the two redox transitions. When the electrode potential is scanned positive, the pTTF/pTTF^+^ redox transition is first brought into “resonance.” At increasingly positive potentials, the second redox transition (pTTF^2+^/pTTF^+^) comes into resonance. Reproduced with permission.^[^ ^56^ ^]^ Copyright 2012, American Chemical Society.

Given the three‐terminal nature of modern electronic devices, establishing a three‐terminal molecular junction employing a single or few molecules as the active component has remained a long‐standing challenge and ultimate goal in the field of molecular electronics. With the development of nanotechnology in the early 21st century, several three‐terminal molecular junction platforms have been reported to use nanogaps produced by electromigration, mechanically controllable break junctions (MCBJs), and STM techniques (Figure 1a). With these techniques, a third terminal based on various gate configurations (e.g., the back gate, side gate, and electrochemical gate) can electrostatically modulate the molecular orbital levels linked between the gaps of the other two terminals.^[^ ^54^, ^55^, ^56^, ^57^, ^74^, ^193^, ^194^, ^195^, ^196^, ^197^, ^198^, ^199^, ^200^, ^201^, ^202^, ^203^ ^]^ These methods have also facilitated the investigation of novel physical phenomena that are difficult to observe in two‐terminal configurations (e.g., the molecular orbital gating effect, Coulomb blockade effect, Kondo effect, and molecular switching effect). For example, Park et al. reported single‐electron phenomena (e.g., the Coulomb blockade and Kondo effect) by using two different molecules composed of Co ions bonded to polypyridyl ligands with different alkyl chain lengths, based on the electromigration nanogap technique (**Figure** **15**a).^[^ ^74^ ^]^ In the junction with longer alkyl chain molecules, the Co ion island was decoupled with both electrodes and its molecular energy level can be tuned using a gate voltage. Therefore, for most values of gate voltage, it is highly probable that the electron will not effectively transfer through the molecular junction, and thus the current will be blocked (Figure 15b). However, the current was abruptly increased when the molecular energy level was matched with Fermi level of electrodes by applying a certain drain and gate voltages (bright line in Figure 15b), that is Coulomb blockade boundary. In contrast, in the junction with shorter alkyl chain molecules, the one energy level with unpaired electron on Co ion island was strongly coupled with the conduction electrons in the metal electrodes; this allowed the electron can be transferred via an exchange process and increase the conductance even at low biases. In addition, the pick split in an applied magnetic field with splitting equal to 2*gµ*_B_ *H* (where *g* ≈ 2, *µ*_B_ is the Bohr magneton, and *H* is magnetic field strength) (Kondo effect) (Figure 15c).

Following this similar method, Song et al. reported the observation of solid‐state molecular field‐effect transistors (FETs) based on the electromigration nanogap technique, in which the direct electrostatic modulation of molecular orbital levels in a three‐terminal solid‐state device configuration was demonstrated (Figure 15d).^[^ ^55^ ^]^ The molecular orbital gating effect was demonstrated using this molecular FET device. In other words, it has been reported that applying a gate electric field can vary the energy offset between the Fermi level of the metal electrode and the molecular orbital levels; this modulates the TVS (Figure 15e). In addition, the resonantly enhanced molecular orbital under gating modulation in the comparison between the near‐ and far‐from‐resonant systems was demonstrated by inelastic electron tunneling (IET) spectroscopy (Figure 15f). In addition, Kay et al. reported the molecular switching characteristics of a redox‐active molecule, pyrrolo‐tetrathiafulvalene (pTTF), based on the STM break junction technique on an applied electrochemical gate field (Figure 15g).^[^ ^56^ ^]^ They demonstrated the ranges of the electrochemical potentials for the pTTF molecules and two redox transitions with a conductance ratio of ≈4 (Figure 15h).

However, these three‐terminal molecular junction platforms suffer from several challenges. Owing to the complexity of the device fabrication processes and the difficulty of obtaining precise 1‐ to 2‐nm‐wide electrode nanogap separation, the electromigration and MCBJ techniques have resulted in a low device yield.^[^ ^27^, ^28^, ^100^ ^]^ Furthermore, the MCBJ technique based on silicon substrates may suffer from breaking of the gating electrode during the bending of the substrate (for separation of the source‐drain electrodes).^[^ ^57^, ^204^ ^]^ To mitigate gate breaking, the electromigration process can be additionally used to aid in the formation of metal nanogaps. In addition to this, a single molecule FET with the MCBJ using nanometer scale noncontact side gate to the molecular junction or using a silicon membrane rather than silicon wafer had been suggested. For the STM‐BJ technique, the gate configuration is often composed of electrochemical gating with ionic liquid, which is useful for studying the charge transport behavior but has limitations for solid‐state molecular device applications.^[^ ^194^, ^197^, ^200^, ^205^ ^]^ Moreover, junction platforms containing single or few molecules are vulnerable to thermal noise and fluctuations, even at room temperature.^[^ ^27^, ^28^, ^100^ ^]^ This places limits upon the operating conditions (e.g., cryogenic temperature or an aqueous environment). To overcome these challenges and issues, a three‐terminal molecular heterojunction with lateral and vertical configuration using a single or bundle of molecules and graphene has recently been suggested as a novel type of molecular transistor.

In this section, we discuss the recently reported three‐terminal molecular heterojunction structures composed of a single molecule or molecular SAMs and graphene, as well as their main electrical characteristics under lateral and vertical configuration. In these device structures, a single (or a few) or molecular SAMs are commonly placed between graphene nanogap or a single layer of graphene (source) and bottom Au (drain) electrodes. A dielectric layer connected to a gate electrode was back‐gate structure or vertically stacked on top of the graphene layer, as shown in Figure 1b.

For the three‐terminal lateral molecule‐graphene heterojunction (left of Figure 1b), the *π*–*π* stacking interaction between molecules and graphene can give stability up to high temperature compared to conventional nanogap‐based molecular junctions, as discussed previously. In addition, since the graphene electrode is extremely thin, the interfacial coupling between molecule and gate field can be enhanced by reducing the screening of an applied gate‐field. Hence, it can highly be likely to exhibit a reliable gate tunable electrical characteristics by modulating the molecular frontier orbital level.

**Figure 16:**
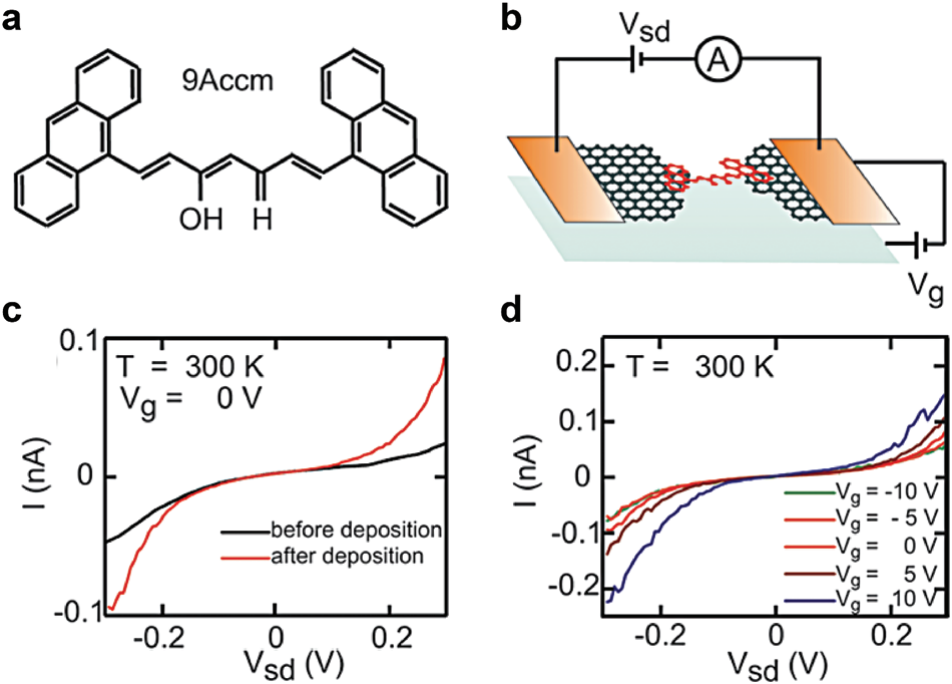
a) Chemical structure of the anthracene‐functionalized curcuminoid molecules (1,7‐(di‐9‐anthracene)‐1,6‐heptadiene‐3,5‐dione). b) Schematics of a single molecule bridging a graphene nanogap. c) *I*–*V* characteristics of the nanogapped electrodes before and after being bridged by molecules at 300 K. d) Dependence of the *I*–*V* characteristics of the nanogapped electrodes bridged by molecules on the applied back‐gate voltage measured at 300 K. Reproduced with permission.^[^ ^206^ ^]^ Copyright 2011, American Chemical Society.

Recently, Prins et al. reported molecular FET devices with nanometer‐separated few‐layer graphene electrodes using feedback‐controlled electroburning technique, and they demonstrated gate‐tunable electrical characteristics at room temperature.^[^ ^206^ ^]^ They formed anthracene‐functionalized curcuminoid molecules (1,7‐(di‐9‐anthracene)‐1,6‐heptadiene‐3,5‐dione) (9Accm) (**Figure** **16**a) on the graphene nanogap devices (Figure 16b). The anthracene‐groups have extended *π*‐conjugated systems that can interact strongly with the top graphene layer, providing strong anchor and high *π*‐electron density that can mediate charge transport in molecule/graphene interface.^[^ ^207^ ^]^ They used few‐layer graphene (3–18 nm) as an electrode to exclude the gating effect of graphene electrode. When the molecular junction formed, the changes in the *I*–*V* characteristics are observed (Figure 16c). According to the gate voltage from −10 and 10 V, the current increased toward more positive gate voltage (Figure 16d). The gate‐tunable characteristics were robust and sustained for periods of several weeks stored in vacuum, and even after thermal cycling to low temperature (10 K). This indicated that graphene nanogap structure can be an alternative to conventional molecular FET devices to overcome the thermal and environmental stability with gate‐tunable electrical characteristics.

For the three‐terminal vertical molecule‐graphene heterojunction (right of Figure 1b), when a gate voltage was applied (via the dielectric layer), the transport barrier at the molecule‐graphene interface could be tuned through the shift of the *E*_F_ for graphene in the electrostatic field. In addition, the unshielded partial electrostatic field achieved by graphene could directly tune the energy level of molecular SAMs or change their redox states, resulting in effective conductance modulation in the device. As a result, in comparison to typical three‐terminal molecular junctions, this three‐terminal vertical molecular heterojunction can be operated reliably at room temperature with a high yield and can also be used to modify the redox state of molecules and the transport barrier in the presence of a gate electric field.

**Figure 17:**
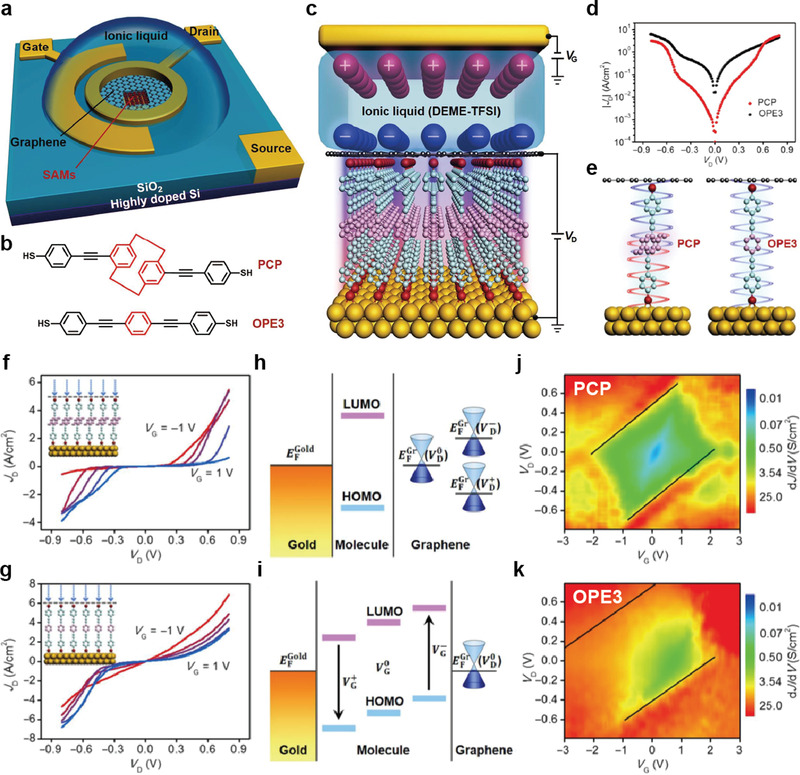
a) Schematic illustration of the overall device structure. b) Chemical structure of the PCP and OPE3 molecules. c) Schematic illustration of the molecular transistor with OPE3 SAMs and ionic liquid (DEME‐TFSI) gating. DEME^+^ ions are the cations, and TFSI^−^ ions comprise the anions. d) Plots of experimental current density (*J*_D_) versus bias voltage (*V*_D_) for PCP and OPE3. e) Schematic illustration of the PCP and OPE3 junctions. *J*_D_ versus *V*_D_ characteristics for f) PCP and g) OPE3 with gating from −1 to 1 V with step of 0.5 V. h) Schematic band diagram of the device under varying *V*_D_ at graphene electrode. i) Schematic band diagram of the device under varying *V*_G_. 2D visualization of d*J*/d*V* plotted with respect to *V*_G_ and *V*_D_ for j) PCP and k) OPT3. Black lines in (j) and (k) are auxiliary markers of corresponding conductance diamond edge. Reproduced with permission.^[^ ^208^ ^]^ Copyright 2018, American Association for the Advancement of Science.

For example, Jia et al. reported molecular vertical tunneling transistors using two different molecular SAMs, and they demonstrated gate‐tunable and stable electrical characteristics at room temperature, as shown in **Figure** **17**a.^[^ ^208^ ^]^ They formed SAMs of pseudo‐*p*‐bis((4‐(acetylthio)phenyl)ethynyl)‐*p*‐[2,2]cyclophane (PCP) or 1,4‐bis(((4‐acetylthio)phenyl)ethynyl)benzene (OPE3) molecules on patterned Au (source)/SiO_2_/highly doped Si substrates (Figures 17a–c). Then, CVD‐grown single‐layer graphene was transferred on top of the SAMs and used as a source electrode. A diethylmethyl(2‐methoxyethyl)ammonium bis(trifluoromethylsulfonyl)imide (DEME‐TFSI) ionic liquid was used as the dielectric layer, and patterned Au (laterally connected to DEME‐TFSI) was used as the gate electrode (Figure 17a,c). The PCP‐graphene heterojunction showed a relatively low conductance near *V*_D_ = 0 compared to the OPE3‐graphene heterojunction (Figure 17d). This is because the PCP molecules exhibit *π*–*π* overlapping between the aromatic rings and methylene bridges; this creates destructive quantum interference features between the HOMO and HOMO‐1 (Figure 17e).^[^ ^209^ ^]^

Figures 17f,g depict the *J*_D_–*V*_D_ curves as the gate voltage (*V*_G_) varies from −1 to 1 V for three‐terminal PCP‐ and OPE3‐graphene heterojunctions, respectively. *V*_G_ can shift both the graphene energy level and molecular orbital level with regard to the *E*_F_ of Au. In other words, when a positive (negative) *V*_G_ is applied, the *E*_F_ of graphene is shifted upward (downward), and the positions of the HOMO and LUMO in the molecular SAMs are shifted downward (upward). When a positive (negative) *V*_D_ is applied under *V*_G_ > 0 (*V*_G_ < 0), the Fermi level approaches the Dirac point of graphene, resulting in the movement of conductance minima in the positive (negative) *V*_D_ direction (Figure 17h,i). Namely, *J*_D_ at positive *V*_D_ decreased when *V*_G_ was changed from −1 V to 1 V, and *J*_D_ at negative *V*_D_ increased when *V*_G_ was changed from −1 to 1 V, regardless of the molecular species (Figure 17f,g). They also found that the gate dependence of the PCP‐graphene heterojunction was much higher than that of the OPE3‐graphene one. As shown in Figure 17j,k, the difference between the low‐conductance region (green) and high‐conductance region (red‐orange) with respect to the gate voltage was much more distinct in the PCP‐graphene heterojunction than in the OPE3‐graphene one. This indicates superior gating tunability for the PCP‐graphene heterojunction, generating a larger on‐off ratio for *J*_D_ at *V*_D_ = 0.

**Figure 18:**
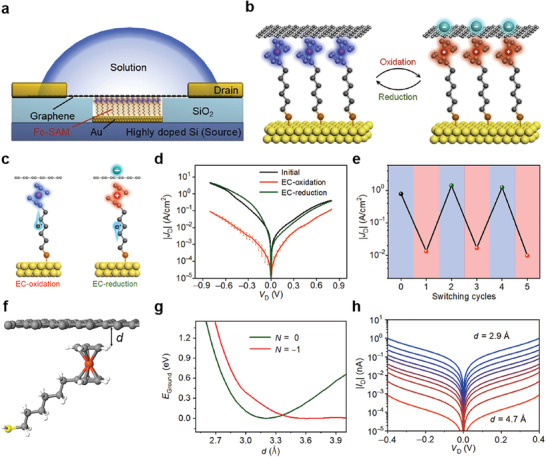
a) Schematic illustration of the device structure of Au/Fc‐SAM/SLG junction with a liquid drop on top of the chip. b) Schematic illustration of the Au/Fc‐SAM/SLG junction with oxidation and reduction treatments. c) Schematic illustration of the EC‐oxidation and EC‐reduction with controlled electron transfer processes. d) *J*_D_–*V*_D_ curves for the junction in initial state, treated by EC‐oxidation and re‐treated by EC‐reduction. The error bars represent the log‐standard deviation. e) Corresponding |*J*_D_| at *V*_D_ = −0.5 V for the junction sequentially treated by EC‐oxidation (red) and reduction (green). f) Schematic representation of the Fc‐graphene contact, where *d* is the contact separation. g) Ground state energy versus *d* for different number of electrons *N* on the molecule: *N* = 0 (neutral) and *N* = −1 (oxidized). h) *I*–*V* characteristics for contact distances of *d* changed from 2.9 to 4.7 Å with step of 0.2 Å. Reproduced with permission.^[^ ^212^ ^]^ Copyright 2022, Elsevier Inc.

In addition to this, using the same molecular vertical transistor configuration, Jia et al. also fabricated a three‐terminal molecular redox‐switching device.^[^ ^210^ ^]^ They formed a monolayer of 6‐ferrocenyl hexanethiol (denoted as FcC_6_S) on an Au/highly doped Si substrate (source) and transferred CVD‐grown single‐layer graphene (drain) on top of the SAM (**Figure** **18**a). The FcC_6_S and gate electrodes were covered with a 0.1 m HClO_4_ solution as the electrolyte solution (Figure 18a). Because FcC_6_S was encapsulated by graphene, the electrochemical redox reactions across single‐layer graphene could be reversibly switched (Figure 18b).^[^ ^211^, ^212^ ^]^ To achieve the electrochemical redox reaction, *V*_G_ was applied to generate an electrochemical double layer on the graphene surface; this can induce electron transfer between the Fc moiety and Au electrode (Figure 18c). In the initial state, an asymmetric *J*_D_–*V*_D_ curve with a maximum RR of ≈14 at ± 0.8 V was observed. When a *V*_G_ of 0.3 V was applied for 180 s, the *J*_D_−*V*_D_ curve was symmetric and its magnitude decreased; this was denoted as an electrochemical (EC) oxidation (Figure 18d). On the other hand, when a *V*_G_ of −0.3 V was applied for 180 s, the *J*_D_−*V*_D_ curve became asymmetric and its magnitude was increased; this was denoted as EC‐reduction (Figure 18d). Figure 18e shows the plot of |*J_D_* | as a function of switching cycles when *V*_G_ was sequentially applied at ±0.3 V for 180 s. The device exhibited reversible switching characteristics and an on/off ratio of two orders of magnitude. A potential gate‐tunable switching mechanism was suggested as follows. Graphene has a high degree of electronic transparency and ion impermeability. Thus, the counter anions can remain separated on the top surface of the graphene layer and balance the oxidized Fc cations under the graphene when a positive *V*_G_ is applied. This forms vertical Fc/graphene or Fc^+^/graphene heterointerface configurations depending on the electrochemical reaction induced by *V*_G_. This reaction can change the distance (*d*) between the graphene and Fc moiety (Figure 18f), by changing the transport width. Figure 18g shows the estimated ground‐state energy (*E*_Ground_) as a function of the contact length (*d*) and *N* (*N* = 0, green line for Fc, and N = −1, red line for Fc^+^). The *d* corresponding to the minimum *E*_Ground_ for N = 0 and −1 was changed from 3.2 to 3.8 Å, affecting the conductance of the molecular junction. Figure 18h shows the calculated *V_D_*−*I_D_* curves for different *d*. They speculated that the lower conductance of the EC oxidation in Figure 18d,e (namely, the Fc^+^ condition) might be due to the increase in *d* under a positive *V*_G_.

To summarize, graphene‐based vertical molecular heterojunction structures offer a novel strategy for fabricating molecular vertical transistors with good stability, reliability, and high yield. In addition, the molecular redox response can be controlled by electrochemical gating effects, to provide a switchable molecular heterojunction platform. Graphene‐based vertical molecular heterojunctions represent promising candidates for molecular‐scale transistors and novel electrical functionalities in molecular‐scale heterojunction structures.

## 4 Prospects and Challenges of Molecular Electronic Heterojunction Devices

This section summarizes and discusses the applicability and merits of molecular heterojunction structures as well as the anticipated challenges and issues associated with their implementation in electronic devices. As mentioned previously, a single molecule or molecular SAMs can create a permanent dipole moment with a strong electric field. This abrupt potential change at the molecular scale can be utilized to tune the energy levels of the metal or semiconductor surfaces.^[^ ^213^ ^]^ Furthermore, this effect can vary depending on the molecular characteristics (e.g., the molecular structure, molecular length, molecular dipole moment, and direction); that is, it could be used to engineer the energy level alignment of subsequent hetero units (e.g., 2D or 3D semiconductors). As a result, it has the potential to control charge transport characteristics in heterojunction systems and generate novel electrical functionalities.^[^ ^84^, ^85^, ^86^, ^87^, ^88^, ^89^, ^90^, ^91^, ^92^, ^93^, ^94^, ^95^, ^96^, ^206^, ^208^ ^]^ Another merit of molecular heterostructures is that there are numerous heterogeneous material combinations and variabilities. Diverse hetero‐units can be combined and included in this junction to implement desirable electronic functionalities. This diversity in hetero units allows the rational design of electronic functions to be extended. These strategies and attempts differ from those of conventional molecular homojunction systems and have the potential to broaden the field of molecular electronics by diversifying the electronic functionalities and novel junction structures. Also, the heterojunction system can significantly improve the device yield and produce excellent operational stabilities, owing to the existence of a subsequent hetero unit after molecular SAMs.^[^ ^84^, ^85^, ^86^, ^87^, ^88^, ^89^ ^]^ This hetero unit effectively prevents the top metal from penetrating the molecular layer, passively and effectively protects the junction from the external environment, and is capable of stabilizing under repeated programming inputs and thermal effects. In addition, those functions and roles of the hetero unit can also increase the degree of integration in both two‐ and three‐terminal vertically stacked molecular heterojunction devices, which can raise the possibility of electronic applications. This is because the molecular SAMs can be encapsulated and protected against environmental factors by hetero‐materials unit, avoiding oxidation and metal penetration under fabrication process. Since the device density can be determined by the cross‐sectional area between the top and the bottom electrode, fabrication methods for decreasing the junction area is necessary. Recently, Kwon et al., made a well‐defined single vertical truncated conical nanopore structure less than 10 nm based on a simple CMOS‐compatible manufacturing process.^[^ ^214^ ^]^ This method for creating a single nanoscale pore could be implemented in vertical two‐ and three‐terminal molecular heterojunction platforms containing a few tens of molecules, thereby significantly increasing device density. In the lateral molecular heterojunctions based on a single molecule and graphene, the ultimate scale of molecular heterojunctions can be realized. Compared to lateral molecular homojunction, the *π*–*π* stacking interaction between molecules and graphene can improve stability up to high temperature. Recently, there is developed a technique that makes uniform nanogap < ≈1 nm between metal on a 4‐in. wafer using the CMOS‐compatible atomic layer lithography technique.^[^ ^215^ ^]^ This technique, which can precisely control the sub‐1 nm gap at the wafer scale, could be used as a platform for FET device for laterally connected molecular heterojunctions. To summarize, these molecular heterojunction systems exhibit considerable potential in terms of tailored electronic functions facilitated by interfacial energy band engineering, numerous heterogeneous material combinations, high device yield, high integration degree, and stability. Given that all of these are related to the long‐standing challenges of conventional solid‐state molecular junction device forms, molecular heterojunction systems have considerable potential for realizing molecular‐scale electronic devices. To summarize various molecular heterojunctions with different hetero units and device structures, we summarized molecules (single or SAM), hetero units, thickness of hetero units, junction scheme, functions, performance, and device yield in **Table** **1**.

**Table 1:** Summary of molecules (single or SAM), hetero units (and its thickness), junction scheme, function, performance, and device yield of molecular heterojunction systems

	Molecule	Single or SAM	Hetero units		Thickness of hetero units	Junction scheme	Function	Performance	Device yield	Ref.
Two‐terminal molecular heterojunction	DTC, C8	SAM	Organic materials	P3HT	100 nm	Crossbar (vertical)	Diode	RR > 2 × 10^3^	N/A	[90]
	2PACz	SAM		PM6:N3	110 nm	Cell (vertical)	Photovoltaic	PCE: 16.60%	N/A	[91]
	C*_n_* (*n* = 8, 10, 12, 14), Diarylethene	SAM		PEDOT:PSS	90–110 nm	via micro hole pattern (vertical)	Flexibility, Photoswitch	ON‐OFF ratio: ≈10	≈90%	[84, 85, 86]
	Nonadiyne	Single	Inorganic materials	Si	Si_HD_ ≈ 1 nm, Si_LD_ ≈ 3 µm	STM (vertical)	Diode	RR > 4 × 10^3^	N/A	[92]
	PDT and 1Ph1	Single		GaAs	GaAs_HD_^:^ ≈ 20 nm, GaAs_LD_: ≈54 nm	STM (vertical)	Photo response	RR > 10^3^	N/A	[93]
	C*_n_* (*n* = 8, 12, 16) and DC8	SAM	2D materials	Graphene	≈10 nm	via micro hole pattern (vertical)	N/A	N/A	≈90%	[87]
	DHA1 and VHF2	SAM		Graphene	≈2 nm	via micro hole pattern (vertical)	N/A	N/A	>70%	[88]
	BDT	SAM		Graphene	Monolayer	EGaIn (vertical)	Thermoelectric	*S* = 30.6 µV K^−1^	N/A	[73]
	C*_n_* (*n* = 4, 6, 8, …, 18)	SAM		Graphene	Monolayer	XPBJ (lateral)	N/A	N/A	N/A	[155]
	PAH	Single		Graphene oxide	Monolayer	via micro hole pattern (vertical)	Photoswitch	ON‐OFF ratio: 5–7	>90%	[89]
	Photochromic azobenzene	SAM		MoS_2_	Monolayer	CP‐AFM (vertical)	Photodiode	ON‐OFF ratio > 10^4^	N/A	[94]
	OPT*_n_* (*n* = 1, 2) and C*_n_* (*n* = 8, 10, 12)	SAM		MoS_2_, WSe_2_	Monolayer–Trilayer	CP‐AFM (vertical)	Diode	RR > 10^3^	N/A	[95]
	C8 and F6H2	SAM		MoS_2_, WSe_2_	Monolayer	CP‐AFM (vertical)	Diode and Selector	RR > 10^2^, ≈482 Gb	N/A	[96]
Three‐terminal molecular heterojunction	9 Accm	Single	2D materials	Graphene	3–18 nm	Nanogap (lateral)	Transistor	ON‐OFF ratio < 10	≈40%	[206]
	PCP and OPE3	SAM		Graphene	Monolayer	via micro hole pattern (vertical)	Transistor	ON‐OFF ratio: ≈320	>90%	[208]
	Fc‐C7	SAM		Graphene	Monolayer	via micro hole pattern (vertical)	Switching transistor	ON‐OFF ratio: ≈140	>90%	[210]

Nonetheless, significant obstacles remain in establishing a genuine molecular electronic heterojunction with desirable electrical and device performances. First, compared with conventional molecular homojunctions, the level of difficulty for device fabrication is significantly increased. Because the fabrication of the molecular heterojunction often requires an additional step to laterally locate the molecules between hetero units, or vertically stack the subsequent hetero unit on or under the molecular SAMs, the fabrication complexity can be further increased; this could lead to unexpected effects such as damage to the molecular layer or non‐uniformity of the heterostructure regions. As the difficulty of fabrication increases, large‐scale mass manufacturing for commercial applications will become more difficult. The second obstacle is the complexity of charge transport mechanisms through the heterostructure. Because the molecular heterojunction system is composed of a combination of a single molecule or molecular SAMs, and organic, inorganic, or 2D nanomaterials, charge transport through these heterostructures may be difficult to comprehend within the standard frameworks typically used for inorganic‐based heterostructures or molecular homojunctions. Given the fact that the transport mechanisms of molecular junction (even for standard alkanethiol molecular junctions) have remained a controversial issue and have been studied by many research groups around the world for decades, it seems very difficult to develop solid theoretical models to fully understand the charge transport properties of various molecular heterojunctions. Third, different technical issues arise, depending on the heterostructure types. For the molecule‐organic heterojunction systems, a second organic layer is typically formed by the spin‐coating process. Owing to the difficulty in filling the organic material via a nanoscale hole, this method may impose constraints on the formation of a nanoscale junction. Furthermore, because the thickness of a coated‐organic layer is on the order of hundreds of nanometers, the effect of molecules in the heterostructure upon the electronic characteristics may be limited. In molecule‐inorganic 3D semiconductor heterojunction systems, molecular SAMs are directly formed on inorganic 3D semiconductors. However, a limited number of molecular anchoring groups are capable of chemically attaching to inorganic semiconductors, which limits the variety of heterostructure combinations. Additionally, because the inorganic hetero unit is positioned beneath the molecules in this type of molecular heterojunction, it is difficult to prevent top metal penetration when fabricating a solid‐state molecular device. This could lead to low device yield and operational instability. Molecular‐2D material heterojunction systems have many strengths in terms of the fabrication of molecular‐scale junctions, implementation of electrical functions, and device yield and stability. However, challenges remain in terms of large‐scale, high‐quality 2D nanomaterial growth and direct transfer onto molecular SAMs. These issues may impede the fabrication of molecular‐scale heterojunction devices on a large scale and may also increase the cell‐to‐cell deviation and junction non‐uniformity. Therefore, the degree of advancements in the growth and fabrication of 2D nanomaterials could significantly affect the development of molecular electronics.

## 5 Conclusion

The field of molecular electronics has seen significant growth in the last half‐century owing to developments in nanofabrication technology and theoretical calculation capabilities. The original goal of implementing a wide variety of electronic functions derived from molecular structures has received significant attention from the perspective of pursuing the ultimate nanoscale electronic devices. Additionally, because molecular building blocks possess infinite degrees of freedom in terms of molecular structure, the growth potential of molecular electronic components remains substantial. Many molecular homojunction platforms have been specially devised and designed to gain a better understanding of the intrinsic charge transport mechanisms via single and molecular SAMs layers, and their electronic device applicability has been thoroughly evaluated. Numerous critical variables governing the electronic behavior and quantum transport in molecular junctions have been identified and investigated. Although our understanding of and ability to fabricate molecular junctions has improved significantly, we are still confronted with significant challenges in implementing a robust molecular‐scale device with desirable electronic functionalities, as mentioned in the introduction. In this review, we introduced novel strategies and methods for overcoming these challenges, in terms of a variety of molecular heterojunction architectures. Diverse molecular heterojunction structures, such as molecule‐organic, molecule‐inorganic semiconductors (Si and GaAs), and molecule‐2D materials (graphene and TMDs) were sequentially presented, along with an explanation of the key parameters governing their electronic functions. As summarized in Section 4, molecular heterojunction systems have unique strengths and merits (compared with typical molecular homojunctions) in terms of their interfacial energy band engineering, countless heterogeneous material combinations, high device yield, and operating stability. With these results, utilizing molecular heterojunction systems may, in our opinion, become a new alternative for validating the promise of molecular devices in commercial electronic applications. Whilst a lot remains to be done before a molecular‐scale electronic device can be realized, we believe that recent advances in molecular heterojunction systems will allow the field of molecular electronics to progress to the next advanced level.

## Conflict of Interest

The authors declare no conflict of interest.
